# DABPU-DE targeting host adipose triglyceride lipase provides broad-spectrum anti-coronavirus effects *in vitro*

**DOI:** 10.3389/fphar.2026.1786048

**Published:** 2026-07-29

**Authors:** Ragab Farouk, Dong Ju Lee, Shaimaa Aboelmagd, Hyung-Jun Kwon, Seong-Hun Jeong, Seok In Han, Dae-Eun Cheong, Sieknay Ny, Hae-Rang Seo, Changhee Lee, Eunae Kim, Sunwoo Lee, Kyoung-Oh Cho

**Affiliations:** 1 Host-Directed Antiviral Research Center, Chonnam National University, Gwangju, Republic of Korea; 2 Department of Avian and Rabbit Medicine, Faculty of Veterinary Medicine, Assiut University, Assiut, Egypt; 3 Department of Zoonoses, Faculty of Veterinary Medicine, Assiut University, Assiut, Egypt; 4 Functional Biomaterials Research Center, Korea Research Institute of Bioscience and Biotechnology, Jeongeup-si, Jeollabuk-do, Republic of Korea; 5 Department of Chemistry, Chonnam National University, Gwangju, Republic of Korea; 6 College of Veterinary Medicine, Gyeongsang National University, Jinju, Republic of Korea; 7 College of Pharmacy, Chosun University, Gwangju, Republic of Korea

**Keywords:** broad-spectrum anti-coronavirus drugs, coronavirus diseases X, DABPU-DM and its derivatives, host-directed antiviral drugs, mutants, SARS-CoV-2

## Abstract

**Objectives:**

This study aims to develop DABPU-DM derivatives that inhibit host adipose triglyceride lipase (ATGL) and identify the one with the highest broad-spectrum anti-coronaviral efficacy.

**Methods:**

The antiviral effectiveness of DABPU-DM derivatives against representative viruses from all four genera within the subfamily *Orthocoronavirinae* was assessed using western blot, RT-qPCR, and immunofluorescence assay (IFA). The reduction in free fatty acid (FFA) and free glycerol (FG) levels following ATGL inhibition was measured with FFA and FG quantification assays. The antiviral effect of DABPU-DE with remdesivir against SARS-CoV-2, either alone or in combination, was evaluated by IFA, and their synergistic effect was analyzed using SynergyFinder 3.0.

**Results:**

Among six ATGL inhibitors, DABPU-DE exhibited the highest antiviral activity and safety, yielding a more favorable selectivity index than the other compounds. The enhanced antiviral effectiveness of DABPU-DE was linked to its diethylurea group, which increases ATGL binding affinity by enhancing hydrophobic and hydrogen-bonding interactions. In contrast, the weakest antiviral effect was observed with DABPU-DPh because its bulky diphenylurea group sterically hindered optimal binding and reduced interactions with ATGL. Additionally, combining DABPU-DE with remdesivir, which targets viral RNA-dependent RNA polymerase, produced strong, synergistic suppression of SARS-CoV-2 infection in Vero E6 cells.

**Conclusion:**

Our findings indicate that the broad-spectrum anti-coronaviral activity of these compounds, particularly DABPU-DE, provides a promising preclinical foundation. This study underscores the potential of ATGL-targeted interventions as a viable strategy for managing current SARS-CoV-2 variants and preparing for future coronavirus outbreaks.

## Highlights

- DABPU-DE has the strongest binding affinity to its host target, ATGL.
- DABPU-DE has excellent broad-spectrum anti-coronavirus effectiveness.
- DABPU-DE could treat current and future coronavirus pandemics.

## 1 Introduction

The COVID-19 pandemic, caused by severe acute respiratory syndrome coronavirus 2 (SARS-CoV-2), has infected over 778 million people and resulted in more than 7 million deaths worldwide as of 2025 (World Health Organization, 2025). Although its exact origin remains unknown, current evidence suggests that all documented human coronaviruses, including SARS-CoV-2, may have evolved from animal hosts such as bats (Cui et al., 2018; Forni et al., 2017; Su et al., 2016; Tang et al., 2022; Zhou et al., 2021). One reason for the emergence of novel coronavirus diseases could be the rapid growth of the human population and increased activity, which have increased encounters with livestock and wild animals, leading to more zoonotic infectious diseases (Jones et al., 2008). Zoonotic diseases account for over 60% of current human infectious diseases and 75% of new or re-emerging ones (WOAH, 2024). Given this history, it is very likely that future outbreaks of unknown coronavirus infections, called Coronavirus Disease X, could also originate in animals.

Compared with many other RNA viruses, coronaviruses—including SARS-CoV-2—tend to exhibit lower mutation rates because their replication machinery includes an nsp14-encoded 3′→5′ exoribonuclease (ExoN) that provides proofreading and increases replication fidelity (Kottur et al., 2022; Minskaia et al., 2006). Nevertheless, even with this proofreading capacity, epidemiologically significant variants can still arise, particularly under conditions of large-scale transmission (which generates vast numbers of replication events), strong or changing selective pressures (e.g., host immunity), and the occurrence of recombination in coronaviruses (Tan et al., 2024; Worobey et al., 2020). This high mutation rate leads to the rapid emergence of variants that may be resistant to current antiviral drugs, such as viral RNA-dependent RNA polymerase (RdRp)-targeting remdesivir and viral 3C-like protease-targeting nirmatrelvir (Hu et al., 2023; Ichikawa et al., 2025). Therefore, it is essential to develop new, advanced drug concepts, such as host-directed, broad-spectrum anti-coronavirus drugs, which can target any SARS-CoV-2 variant and future emerging coronaviruses, such as the predicted Coronavirus Disease X (Cakir et al., 2021; Kumar et al., 2020; Meganck and Baric, 2021; Scheriber and Ludwig, 2025).

Since viruses lack their own replication machinery, they hijack host cell resources to complete their life cycle. Lipids and lipid droplets (LDs) are essential in this process. By manipulating them, viruses acquire the resources and environment needed for efficient replication, assembly, and immune evasion, such as forming viral morphogenesis, serving as energy sources, and contributing to eicosanoid and cytokine storms (Cesar-Silva et al., 2023; Crawford and Desselberger, 2016; Criglar et al., 2022; Huitron et al., 2023; Monson et al., 2021a; Qu et al., 2023). Coronavirus-induced LDs in virus-infected cells serve as hubs for lipid storage and signaling that support the membrane and energy demands of viral replication (Dias et al., 2020; Snijder et al., 2020; Klein et al., 2020). Studies on SARS-CoV-2 show that infection promotes LD biogenesis and that disrupting LD formation or lipid mobilization can reduce viral replication, indicating LD pathways are functionally pro-viral (Dias et al., 2020; Nardacci et al., 2021). More broadly, coronaviruses rely on host lipid synthesis programs (including SREBP-dependent lipogenesis) that feed replication-organelle membrane biogenesis and lipid storage/LD dynamics (Yan et al., 2019; Yuan et al., 2019; Klein et al., 2020). LD remodeling has also been linked to the production of inflammatory mediators during infection, coupling viral replication with pathogenic host responses (Dias et al., 2020).

Host enzymes involved in lipid and LD formation and breakdown are promising targets for antiviral drugs. For example, TOFA and C75, inhibitors of Acetyl-CoA Carboxylase (ACC) and Fatty Acid Synthase (FAS), respectively, disrupt fatty acid biosynthesis and impair the replication of various viruses, including rotavirus, Enterovirus A71, West Nile virus, and MERS-CoV (Gaunt et al., 2013; Yang et al., 2022; Merino-Ramos et al., 2015; Soultsioti et al., 2025). Sterol regulatory element-binding proteins (SREBPs), which are bHLH-zip transcription factors, play vital roles in maintaining cellular lipid balance (Yuan et al., 2019). Notably, AM580 inhibited the replication of several viruses, including MERS-CoV, influenza A virus (IAV), and Enterovirus A71, by binding to SREBP1 and SREBP2 (Yuan et al., 2019). Inhibitors targeting LD-associated lipases, such as Atglistatin (DABPU-DM) and CAY10499, have been shown to block viral replication *in vitro* and *in vivo* (Blázquez et al., 2025; Laufman et al., 2019).

In this study, we developed five derivatives of Atglistatin (DABPU-DM), an inhibitor of adipose triglyceride lipase (ATGL)—a host lipid droplet-associated lipase essential for viral replication (Blázquez et al., 2025; Laufman et al., 2019). We evaluated the safety and antiviral efficacy of these compounds against representative coronaviruses from all four genera of the subfamily *Orthocoronavirinae*. Our findings demonstrate that the broad-spectrum antiviral activity of these derivatives, particularly DABPU-DE, establishes a promising early-stage preclinical platform for ATGL-targeted, host-directed interventions to address current SARS-CoV-2 variants and mitigate future coronavirus pandemics.

## 2 Materials and methods

### 2.1 Cells and viruses

African green monkey kidney epithelial Vero E6, human lung adenocarcinoma Calu-3, human colorectal adenocarcinoma HRT-18G, and swine testicular (ST) cells were obtained from the American Type Culture Collection (ATCC, Manassas, VA, USA). Chicken embryo kidney (CEK) cells were prepared using 18-day-old specific pathogen-free chick embryos, as described elsewhere (Lee et al., 2021). They were cultured in Dulbecco’s Modified Eagle’s Medium (DMEM) or alpha Minimum Essential Medium (α-MEM), supplemented with 10% fetal bovine serum (FBS), 100 U/mL penicillin, and 100 μg/mL streptomycin, at 37 °C in a humidified atmosphere with 5% CO_2_ for 3–4 days (Supplementary Table S1).

Human lung organoids (SNU-2637-CO) from a patient with lung adenocarcinoma obtained from the Korean Cell Line Bank (Seoul, Korea) were cultured as described elsewhere (Castaneda et al., 2023; Ekanger et al., 2022). Single human lung organoid stock cells were resuspended with advanced DMEM and centrifuged at 200 g for 5 min at 4 °C. After removal of the supernatant, organoid cell pellets were resuspended with lung organoid media supplemented with 100 U/mL penicillin, and 100 μg/mL streptomycin, added with the same amount of Matrigel (Sigma Aldrich, MO, USA), and then cultured at 37 °C in a humidified atmosphere with 5% CO_2_ for 2 weeks (Supplemntary Table S2).

The porcine epidemic diarrhea virus (PEDV) QIAP1401 strain was a kind gift from the Animal and Plant Quarantine Agency (APQA) of Korea (Gimcheon, South Korea) and propagated in Vero E6 cells (Lee et al., 2015). Four strains of SARS-CoV-2, KCDC03 (isolated from a Korean COVID-19 patient in 2020 and belonging to the A lineage with early Chinese strains), KDCA51463 (isolated from a Korean COVID-19 patient in 2021 and belonging to the Alpha lineage), KDCA55905 (isolated from a Korean COVID-19 patient in 2021 and belonging to the Beta lineage), and NCCP43426 (isolated from a Korean COVID-19 patient in 2022 and belonging to the Omicron BA.5 lineage) stains were kindly provided by the Korea Disease Control and Prevention Agency (Cheongju, South Korea) (Konings et al., 2021) and propagated in Vero E6 cells. The bovine coronavirus (BCoV) KWD20 strain was isolated from a fecal sample of an adult cow with diarrhea and propagated in the human rectal tumor cell line HRT-18G (Park et al., 2006). Infectious bronchitis virus (IBV) Kr/D49/20 strain was kindly provided by AQPA (Gimcheon, South Korea) and propagated in CEK cells (Lee et al., 2021). Porcine deltacoronavirus (PDCoV) KOR/KNU16-07 strain was kindly provided by the APQA (Gimcheon, South Korea) and propagated in ST cells (Lee et al., 2023). Information on virus strains and culture conditions is provided in the Supplementary Material (Supplementary Tables S3, S4).

### 2.2 Reagents, antibodies, and kits

DABPU-DM (Atglistatin) and remdesivir — both purchased from MedChem Express (Monmouth Junction, NJ, USA) — along with five derivatives of DABPU-DM (DABPU-DE, DABPU-EM, DABPU-DPh, DABPU-Pyrrol, and DABPU-Morph) generated in this study were dissolved in an N, N-dimethylacetamide (DMA)-based solvent consisting of 20% (v/v) DMA, 60% (v/v) polyethylene glycol 400, 2% phosphoric acid, and phosphate-buffered saline (PBS, pH 7.4) for *in vitro* and *in vivo* experiments. DABPU-DE was also dissolved in an N-methyl-2-pyrrolidone (NMP)-based solvent containing 10.67% (v/v) NMP, 2.4% (v/v) phosphoric acid, 1.5% (w/v) KOH, and PBS (pH 7.4) for *in vitro* and *in vivo* experiments (Supplementary Table S5). Saturated palmitic acid (PA), monounsaturated oleic acid (OA), and polyunsaturated linoleic acid from Sigma Aldrich were dissolved in PBS (pH 7.4). BODIPY (493/503), 3-(4,5-dimethylthiazol-2-yl)-2,5-diphenyl tetrazolium bromide (MTT), Triton X-100, and Matrigel were purchased from Sigma Aldrich. Slow-Fade Gold antifade reagent and paraformaldehyde were obtained from Molecular Probes (Eugene, OR, USA) and Sigma Aldrich, respectively. The RNeasy Mini Kit and the BCA protein assay kit were purchased from Qiagen (Hilden, Germany) and Thermo Scientific, respectively. The free fatty acid (FFA) quantification kit, free glycerol quantification kit, and lactate dehydrogenase (LDH) assay kit were purchased from Abcam (Cambridge, MA, USA); Lipofectamine 3,000, Alexa Fluor™ 488 Phalloidin were obtained from Thermo Scientific (Waltham, MA, USA); and the SensiFAST SYBR Lo-ROX One-Step Kit was purchased from Meridian Bioscience (Cincinnati, OH, USA).

Primary antibodies used in this study include a rabbit polyclonal antibody (Pab) against the SARS-CoV-2 spike (S) protein and a mouse monoclonal antibody (Mab) against the SARS-CoV-2 nucleocapsid (N) from Abcam (Cambridge, UK) and Cell Signaling (Beverly, MA, USA), respectively. Additionally, BCoV N Mab from Abcam, PEDV N Mab from Medgene Labs (Brookings, SD, USA), IBV N Mab from Novus (Centennial, CO, USA), PDCoV N Pab from Humimmu (Salem, NH, USA), GAPDH from Santa Cruz (Dallas, TX, USA), and ATGL from Cell Signaling (Beverly, MA, USA) were used. The secondary antibodies consist of Alexa Fluor 594 (AF594) and AF488-labeled donkey Pabs against rabbit immunoglobulin G (IgG), as well as AF594- and AF488-labeled goat Pabs against mouse IgG, all sourced from Thermo Scientific. Horseradish peroxidase (HRP)-conjugated secondary antibodies, including goat anti-rabbit IgG from Cell Signaling and goat anti-mouse IgG from Santa Cruz, were used for the Western blot. siRNAs were purchased from Bioneer Inc. (Daejeon, South Korea). The reagents, kits, and antibodies employed in this study are listed in Supplementary Tables S6, S7.

### 2.3 Ethics statement

All animal experiments were performed in accordance with the guidelines and requirements of the Institutional Animal Care and Use Committee at Chonnam National University (CNU IACUC-YB-2024-01). Animal care and handling complied with current international laws and policies, including the NIH Guide for the Care and Use of Laboratory Animals (NIH Publication No. 85–23, 1985; revised 1996). Every effort was made to minimize the number of animals used and to reduce animal suffering.

### 2.4 Experimental animals

Seven-week-old female wild-type Sprague–Dawley rats were purchased from Samtako (Osan, South Korea). Animals were housed in standard cages with *ad libitum* access to food and water in a specific-pathogen-free facility under controlled conditions (12-h light/12-h dark cycle, 25 °C, and 40%–55% relative humidity). After a 1-week acclimatization period, the rats were used for the experiments described below.

### 2.5 *In vivo* toxicity test

To evaluate the safety profile of DABPU-DE, rats were randomly allocated to four experimental groups (n = 5 per group), with the sample size determined based on our previous studies to provide sufficient statistical power (1-β > 0.8). For the NMP-based formulation study, animals were anesthetized with isoflurane (Terrell solution, 1.5% v/v) administered via a gas-evacuation apparatus (R546Pro, RWD Life Science, TX, USA). The control group received 100 μL of vehicle [10.67% N-methyl-2-pyrrolidone (NMP), 2.4% phosphoric acid (PA), 1.5% potassium hydroxide (KOH), and 75% phosphate-buffered saline (PBS)] via tail vein intravenous (IV) injection once daily for 5 consecutive days. The treatment groups received DABPU-DE intravenously at doses of 1, 5, or 10 mg kg^−1^·day^−1^ for the same duration. Animals were monitored daily for 14 days for clinical abnormalities—including neurological, gastrointestinal, and respiratory signs—and mortality. An identical study design and dosing regimen were employed for intramuscular (IM) administration, with injections delivered into the biceps femoris.

Similarly, to assess the toxicity of DMA-based formulations, rats (n = 5 per group) were randomly assigned to receive either an IV or an IM injection. The control group received a vehicle consisting of 20% DMA, 60% polyethylene glycol 400 (PEG400), 2% PA, and 10% PBS. Treatment groups (Groups 2–4) received DABPU-DE (20 mg kg^−1^·day^−1^) in modified vehicles with varying concentrations of PA (Group 2: 1.0% PA; Group 3: 1.5% PA; Group 4: 2.0% PA). Following the 14-day observation period, all animals were euthanized via cervical dislocation under anesthesia. Major organs, including the lungs, liver, kidneys, heart, spleen, and the biceps femoris at the injection site, were harvested for gross and histopathological examination.

### 2.6 Histopathology

Histopathological evaluations were conducted as previously described. Briefly, harvested tissues were fixed in 10% neutral buffered formalin at 20 °C for 3–4 days, dehydrated through a graded ethanol series, and embedded in paraffin using an automated processor. Sections (3 μm) were prepared using a microtome and stained with hematoxylin and eosin (H&E). Importantly, morphological assessments were conducted in a blinded manner; the pathologists and investigators were unaware of the specific group assignments until the final data analysis stage, thereby eliminating observer bias.

### 2.7 Synthesis of atglistatin and its derivatives

The derivatives of DABPU-DM were synthesized through acyl substitution reactions between N4′, N4′-dimethyl- [1, 1′-biphenyl]-3,4′-diamine—prepared via Suzuki coupling, followed by reduction, and various carbamoyl chlorides with different substituents.

### 2.8 Viral titration

The viral titers for PEDV, SARS-CoV-2, BCoV, IBV, and PDCoV were measured using a cell culture immunofluorescence (CCIF) assay and are expressed as fluorescence focus units per milliliter (FFU/mL). For viral titration, monoclonal antibodies specific to the SARS-CoV-2 spike (S) protein, PEDV nucleoprotein (N), BCoV N protein, PDCoV N protein, and IBV N protein are used (Supplementary Table S7).

### 2.9 Determination of 50% cytotoxicity concentration (CC_50_)

The CC_50_ of the tested chemicals was measured using the MTT assay to evaluate cell viability, as described elsewhere (van Meerloo et al., 2011). Briefly, 50 mM stock solutions of each chemical were prepared in a DMA-based solvent and then serially diluted in serum-free medium. These dilutions were applied to monolayers of each corresponding cell type. Absorbance was measured at 570 nm using an enzyme-linked immunosorbent assay (ELISA) reader. Percent cell viability was calculated with the formula: [(OD sample – OD blank)/(OD control – OD blank)] × 100%. Dose-response curves were generated from the cytotoxicity data, and CC_50_ values were determined using GraphPad Prism software (version 10.4.1).

### 2.10 LDH assay

LDH release, as an indicator of membrane damage, was quantified in each cell type after treatment with the test compounds using a commercially available Cytotoxicity Detection Kit (LDH; ab102526, Abcam, Cambridge, UK). After treatment, culture plates were centrifuged at 400 × g for 4 min at 4 °C. The culture supernatant was diluted, and 50 µL was transferred to a fresh 96-well plate for LDH measurement. The assay was conducted according to the manufacturer’s protocol, and absorbance was read at 450 nm using a microplate reader.

### 2.11 Determination of broad-spectrum antiviral effects

The broad-spectrum antiviral effects of each small molecule were evaluated as described previously. Briefly, each cell line—Vero E6 for PEDV, Vero E6 and Calu-3 for SARS-CoV-2, HRT-18G for BCoV, CEK for IBV, and ST for PDCoV—was grown to 80% confluence in a 6-well plate. The cells were then infected with a multiplicity of infection (MOI) of 0.1 or 0.2 FFU or left uninfected as controls. After a 1-h virus absorption period, the cells were washed twice with Dulbecco’s phosphate-buffered saline (DPBS) and refreshed with fresh DMEM containing 100 U/mL penicillin and 100 μg/mL streptomycin. DABPU-DM and its derivatives were treated with various concentrations of each small molecule or vehicle for specified incubation times (Supplementary Table S8). Finally, the cells were harvested to quantify viral expression levels using a CCIF assay as described below.

### 2.12 Transfection of siRNA

Vero E6 cells were seeded in 6-well culture plates or 8-well chamber slides and grown to 70%–80% confluence. Cells were transfected with siRNA targeting ATGL (10 nmol) using Lipofectamine 3,000 (Thermo Fisher Scientific) as described previously (Alfajaro et al., 2019). To enhance knockdown efficiency, a second transfection was performed 24 h after the initial transfection. Scrambled siRNA (10 nmol) was transfected in parallel as a negative control. Subsequently, cells were infected with SARS-CoV-2 (KCDC03, lineage A) at an MOI of 0.1. After a 1-h adsorption period, cells were washed three times with DPBS and maintained in fresh DMEM supplemented with 100 U/mL penicillin and 100 μg/mL streptomycin. Cells cultured on chamber slides were fixed at 36 h post-infection (hpi) to assess LD retention and SARS-CoV-2 antigen expression. Cells in 6-well plates were harvested at 36 hpi for, viral protein analysis by western blotting, and viral genome quantification by RT–qPCR.

### 2.13 Virus rescue assay

We examined whether viral replication resumes when FFAs are added to virus-infected cells deficient in FFAs following DABPU-DE treatment as described previously. In brief, confluent Vero E6 cells were infected with the SARS-CoV-2 KCDC03 strain at an MOI of 0.1 FFU and then vehicle-treated, treated with 10 µM of DABPU-DE at the middle stage (18 hpi) of infection, or DABPUE-DE treated at the middle stage (18 hpi) supplemented with 100 μM palmitic acid, oleic acid, or linoleic acid and incubated for an additional 18 h. After three cycles of freezing and thawing, serial 10-fold dilutions of each cell culture supernatant were prepared in EMEM supplemented with 100 U/mL penicillin and 100 μg/mL streptomycin. Viral genome copy numbers and titer were determined by cell culture immunofluorescence (CCIF) assay and reverse transcription quantitative polymerase chain reaction (RT-qPCR) as described below.

### 2.14 Immunofluorescence assay (IFA)

To characterize the dynamics of LD formation and the inhibitory effect on viral protein expression for each small molecule, an IFA was performed using Vero E6, Calu-3, ST, HRT-18G, and CEK cells either infected with or without the corresponding virus and treated with vehicle or chemical (Kim et al., 2010; Monson et al., 2021b; Soliman et al., 2022). Briefly, each cell line grown to 80% confluence in wells of a 6-well plate, an 8-well chamber slide, or a 96-well plate was infected with an MOI of 0.1 or 0.2 FFU, or left mock-infected with medium. Next, each well was treated with 10 μM of each small molecule, either immediately after virus absorption or at mid-stage of infection, and then incubated for a fixed period (Supplementary Table S8).

The antiviral effects of DABPU-DE in human lung organoids were determined as described elsewhere (Martinez-Ordoñez et al., 2023; Yuan et al., 2019). In brief, Matrigel domes containing organoids grown in a 6-well plate for 2 weeks were melted with PluriSTEM® Dispase-II Solution and incubated for 1 h. After centrifugation at 200 g for 5 min at 4 °C and removal of the supernatant, organoid pellets were infected with the SARS-CoV-2 KCDC strain in 100 μL of organoid basal medium at an MOI of 0.5 FFU, or left mock-infected. To promote efficient infection of organoid cells, organoid clumps were disrupted by 20 pipetting cycles to promote viral attachment to the apical surface, then incubated for 1 h to allow viral absorption. After washing with 200 μL DPBS, the mixture was centrifuged at 200 g for 5 min at 4 °C. After removal of the supernatant, the organoid cells were resuspended in 200 μL Matrigel, and 100 μL of each was transferred into one well of an 8-well chamber slide. To promote efficient delivery of DABPU-DE to infected organoid cells, organoid clumps were disrupted by 20 pipetting cycles, then treated with 10 μM DABPU-DE at 12 hpi, and then incubated for an additional 12 h (Supplementary Table S8).

The cells were fixed with 4% buffered paraformaldehyde (PFA) for 10 min (cultured cells) or 30 min (organoids) at 20 °C, washed twice with DPBS, and permeabilized with 0.2% Triton X-100 for 10 min (cultured cells) and 1 h (organoids) at 20 °C. Primary antibodies against each target cellular or viral protein were applied overnight at 4 °C, then washed three times with PBS (pH 7.4) containing 0.1% newborn calf serum (PBS-NCS). Secondary antibodies conjugated with Alexa Fluor 488 (AF488) or AF594 were incubated for 1 h at 20 °C. To assess LD formation, selected slides were further incubated with 10 μM BODIPY 493/503 for 10 min at 20 °C. Afterward, cells were washed three times with PBS (pH 7.4) and mounted with SlowFade Gold antifade reagent containing 1× DAPI solution (Molecular Probes) for nuclear staining. Finally, they were imaged using an LSM 800 confocal microscope and analyzed with Zen Blue Software 2.6 (Carl Zeiss; Jena, Germany).

The antiviral effects of each small molecule and virus rescue assay were assessed using the CCIF assay. Briefly, the 6-well plates prepared as described above underwent three freeze-thaw cycles. Serial 10-fold dilutions of each cell culture supernatant were prepared in a 96-well plate with DMEM supplemented with 100 U/mL penicillin and 100 μg/mL streptomycin. One hundred microliters of each tenfold dilution were transferred in triplicate to Vero E6, Calu-3, HRT-18G, CEK, or ST cells, which were cultured in each well of the 96-well plates. The cells were then incubated for 18 h at 37 °C in a CO_2_ incubator, fixed with 80% cold acetone, and washed twice with PBS (pH 7.4). Antibodies targeting PEDV N, SARS-CoV-2 S, BCoV N, IBV N, or PDCoV N protein were applied to the respective well. After washing twice with PBS (pH 7.4), each well was incubated for 1 h at 37 °C with the secondary antibody conjugated to AF488 or AF594. The cells were then washed twice with PBS (pH 8.0), mounted in 60% glycerol, and examined using inverted fluorescence microscopy. The number of cells showing a viral antigen-positive signal at the endpoint dilution was counted.

The half-maximal inhibitory concentration (IC_50_) of DABPU-DM, its derivatives, and remdesivir was determined using an IFA as described above, with slight modification. The differences are that the cells were treated with various drug concentrations under the above conditions, and propidium iodide (PI) was added at 37 °C for 1 h to stain the nuclei. Afterward, the cells were washed twice with PBS (pH 8.0) and mounted with 60% glycerol. Fluorescence intensities in each well, indicating viral positive signals, were measured using a fluorescence reader with the following formula: [(Green fluorescence intensity/Red fluorescence intensity by PI in virus-infected, chemical- or vehicle-treated target group)/(Green fluorescence intensity/Red fluorescence intensity of PI in mock-infected, vehicle-treated control group)] × 100%. The percentage of inhibition was calculated as 100 minus the infectivity percentage. IC_50_ was determined using GraphPad Prism software version 10.4.1. The Selectivity Index (SI) of the chemicals was calculated as CC_50_ divided by IC_50_.

### 2.15 FFA quantification

A FFA Quantification Kit (Abcam) was used to measure the amount of intracellular FFAs in Vero E6, Calu-3, HRT-18G, CEK, and ST cells infected with or without each virus or treated with or without different concentrations of chemicals, as described elsewhere (Lee et al., 2017; Zhao et al., 2024). In brief, 5 × 10^6^ cells were mixed with 200 μL of chloroform-Trition X-100 (1% Triton X-100 in pure chloroform) and vortexed every 5 min for 20 min at 20 °C. The organic phase in the lower layer was collected, and chloroform was air-dried for 20 min at 60 °C. The dried lipids were then dissolved in 200 μL of Fatty Acid Assay Buffer by vortexing for 5 min at 20 °C, and 50 μL of the supernatant was transferred to a 96-well plate along with serially diluted standard samples. Each well received 2 μL of ACS Reagent and was incubated at 37 °C for 30 min. Subsequently, all samples and standards were treated with 50 μL of Reaction Mixture (Enzyme Mix and Enhancer) and incubated at 37 °C for an additional 30 min. Measurement was performed by determining the OD at 570 nm using an ELISA reader. Calculations were then carried out, followed by the generation of a standard curve according to the manufacturer’s instructions.

### 2.16 Free glycerol quantification

The amount of intracellular free glycerol in Vero E6, Calu-3, ST, HRT-18G, and CEK cells infected with or without each virus or treated with or without different concentrations of chemicals was measured using a quantification kit according to the manufacturer’s recommendations (Cimas et al., 2023; Siderius et al., 2000). In brief, 5 × 10^6^ cells were mixed with 500 μL of Glycerol Assay Buffer and vortexed at 5-min intervals for 20 min at 20 °C. After centrifugation, 50 μL of the supernatant was transferred to a 96-well plate along with the serially diluted glycerol standard samples. Then, 50 μL of Reaction Mixture, which includes the Enzyme Mix and Glycerol Probe, was added and incubated at 20 °C for 30 min. The measurement was performed by reading the OD at 570 nm using an ELISA reader. Calculations were carried out, and a standard curve was generated according to the manufacturer’s instructions.

### 2.17 Quantitative real-time PCR

Vero E6, Calu-3, ST, HRT-18G, and CEK cells infected with the corresponding virus or mock-infected and treated with various chemical concentrations (Atglistatin and its derivatives and FFAs) or vehicle, as described above, were used to extract total RNA. The host mRNA and viral genome copies were quantified by real-time PCR as previously described, with minor modifications (Jeon et al., 2020; Liu et al., 2022). Briefly, total RNA was extracted using the RNeasy kit (Qiagen) following the manufacturer’s instructions. The corresponding cDNA was synthesized using the SensiFAST SYBR Lo-ROX one-step kit (Meridian Biosciences). Each 20 μL reaction mixture contained 10 μL of SensiFast Sybrgreen, 0.8 μL of forward primer, 0.8 μL of reverse primer, 0.2 μL of reverse transcriptase, 0.4 μL of RiboSafe RNase inhibitor, 5 μL of template, and 2.8 μL of DEPC-treated water. A specific primer pair is listed in Supplementary Table S9. The LineGene 9,600 Plus Real-time PCR system (Bioer, Hangzhou, China) was used with the following conditions: for viral genome copy number, activation of DNA polymerase at 95 °C for 10 min, then 40 cycles of three steps at 95 °C for 15 s, 60 °C for 20 s, and 72 °C for 20 s; for cellular target gene mRNA, activation of DNA polymerase at 95 °C for 10 min, then 40 cycles of three steps at 95 °C for 15 s, 55 °C for 20 s, and 72 °C for 20 s. The reduction in viral load was expressed as relative values, with genome copy numbers in virus-infected, chemically treated groups normalized to those in the virus-infected, mock-treated control. GAPDH served as an endogenous reference gene to normalize target gene expression levels. Relative quantification of gene expression was determined using the 2^−ΔΔCt^ method following normalization against GAPDH (Livak and Schmittgen, 2001).

### 2.18 Cellular thermal shift assay (CETSA)

Target engagement of DABPU-DE was evaluated using the cellular thermal shift assay (CETSA) as previously described (Jafari et al., 2014; Hashimoto et al., 2018). Briefly, Vero E6 cells were treated with either 10 µM DABPU-DE or vehicle for 1 h. Following treatment, cells were washed with PBS, harvested, and lysed using a specialized lysis buffer. The cell lysates were clarified by centrifugation at 14,000 × g for 20 min at 4 °C. The resulting supernatants were divided into equal aliquots in PCR tubes and subjected to a temperature gradient ranging from 40 °C to 63 °C for 3 min using a thermal cycler, followed by incubation at room temperature for 3 min. The heated samples were then centrifuged at 14,000 × g for 20 min to pellet the denatured and precipitated proteins. The remaining soluble fractions were collected and analyzed via immunoblotting to assess the thermal stability of the target protein.

### 2.19 Total cellular lipase activity assay

Intracellular lipase activity was quantified using a Lipase Assay Kit (Sigma-Aldrich) following the manufacturer’s protocol. In brief, confluent Vero E6 cells were infected with the SARS-CoV-2 KCDC03 strain at an MOI of 0.1 FFU and then vehicle-treated, treated with 10 µM of DABPU-DE or CAY10499 at the middle stage (18 hpi) of infection, incubated for an additional 18 h, and underwent three freeze-thaw cycles. The harvested cells were homogenized in 200 µL of ice-cold 1× PBS. The homogenates were centrifuged at 14,000 × g for 5 min at room temperature to remove cellular debris. A 10-µL aliquot of the resulting supernatant was transferred to a 96-well microplate and mixed with 140 µL of the reaction mixture. Serial dilutions of standards and appropriate blank controls were processed in parallel. Following incubation at room temperature for the specified duration to facilitate enzymatic hydrolysis of the substrate, the absorbance of the chromogenic product was measured at 412 nm using a microplate reader.

### 2.20 Western blot analysis

The expression levels of cellular and viral proteins were determined by Western blot analysis as previously described (Aboelmagd et al., 2026). Briefly, cells were lysed on ice for 10 min using RIPA buffer containing 10 mM Tris-HCl (pH 7.4), 100 mM NaCl, 1 mM EDTA, 1 mM EGTA, 1 mM NaF, 20 mM Na_2_P_2_O_7_, 2 mM Na_3_VO_4_, 1% Triton X-100, 10% glycerol, 0.1% SDS, and 0.5% sodium deoxycholate (Invitrogen). Cell lysates were centrifuged at 12,000 × g for 10 min at 4 °C, and the supernatants were collected for protein quantification using a BCA protein assay kit (Thermo Scientific). Equal amounts of protein were separated by SDS–PAGE and transferred onto nitrocellulose membranes (GE Healthcare Life Sciences). Membranes were blocked for 1 h at room temperature in Tris-buffered saline containing 5% skim milk and 0.1% Tween-20, then incubated with the appropriate primary antibodies overnight at 4 °C with gentle agitation. After washing, the membranes were incubated with HRP-conjugated secondary antibodies for 1 h at room temperature. Protein bands were visualized using an enhanced chemiluminescence (ECL) detection system (Dogen, Seoul, South Korea) and imaged using a Davinch-K Imaging System (Youngwha Scientific Co., Ltd., Seoul, South Korea). Band intensities were quantified and normalized to GAPDH as a loading control, and the results were expressed as fold changes relative to the corresponding control group.

### 2.21 Synergistic effect of combination treatment of DABPU-DE and remdesivir

In 96-well plates, Vero E6 cells were infected with SARS-CoV-2 KCDC03 at an MOI of 0.1. The cells, either mock-infected or virus-infected, were treated with vehicle or the different concentrations of remdesivir immediately after virus absorption and incubated for 36 h. Various concentrations of DABPU-DE were treated to virus-infected cells at 18 hpi and incubated for an additional 18 h. In the combination group of DABPU-DE and remdesivir, infected cells were treated with various remdesivir concentrations immediately after virus adsorption, followed by different DABPU-DE concentrations at 18 hpi, and incubated for an additional 18 h. At 36 hpi, cells were fixed with cold 80% acetone for further processing and assessment of the inhibitory effect by IFA, as described above.

### 2.22 Analysis of the synergistic effect of combining DABPU-DE and remdesivir to inhibit SARS-CoV-2

The synergistic effects of combining DABPU-DE and remdesivir to inhibit SARS-CoV-2 were analyzed using SynergyFinder 3.0 (https://synergyfinder.org). Data points were excluded based on the results from different concentrations of DABPU-DE and remdesivir, whether alone or in combination: (1) complete inhibition of virus infection (100%) or (2) inhibition less than 25% (i.e., insufficient antiviral activity against SARS-CoV-2) (Chou, 2014; ComboSyn Inc., 2021; Doki et al., 2022). The drug interactions were assessed by calculating the combination index (CI) (Chou, 2006; Chou and Talalay, 1984; Doki et al., 2022). The CI values were derived using these equations (Chou and Talalay, 1984; Doki et al., 2022): [(Dose of drug one in the combination that produces x % inhibition/dose of drug one alone that causes the same x % inhibition) + (Dose of drug two in the combination that produces x % inhibition/dose of drug two alone that causes the same x % inhibition)]. The Fa (fraction affected) values were calculated using the equations (Chou and Talalay, 1984): (percent inhibition/100). The Fa–CI plot was created with GraphPad Prism version 10.4.1. 4. 1 to illustrate drug combination effects, where values indicate synergism (CI < 1), additivity (CI = 1), or antagonism (CI > 1). Further analysis was performed with SynergyFinder 3.0 (https://synergyfinder.org). Dose–response curves were fitted using a four-parameter log-logistic (LL4) regression model with baseline correction to account for background signal. Synergy was quantified using the Bliss independence reference model, for which the expected combined effect is defined as y BLISS = y1 + y2 − y1.y2. In addition, we also used different synergistic models to evaluate synergism, such as ZIP, HSA, and Loewe.

Bliss scores >10, <−10, and between −10 and 10 were interpreted as synergistic, antagonistic, and additive effects, respectively. Local synergy was further evaluated using the Most Synergistic Area (MSA) score, defined as the highest Bliss score calculated within a 3 × 3 region of the dose matrix. Each drug combination was evaluated in three independent biological experiments, each performed in technical triplicate. Data were imported into SynergyFinder 3.0 in the replicate-example format to ensure appropriate curve-fitting and synergy analysis (Zhao et al., 2014; Wagoner et al., 2022).

### 2.23 Computational target protein modeling and molecular docking

To elucidate the molecular interactions between human ATGL and its inhibitors, the patatin domain (residues 10–178) of the ATGL structure (UniProt: Q96AD5) was retrieved from the AlphaFold database (Jumper et al., 2021). The structure was refined using the GROMACS package (Abraham et al., 2015) with the AMBER99SB force field (Hornak et al., 2006). The system was solvated in a cubic box using the TIP3P water model (Jorgensen et al., 1983) and neutralized with eight chloride ions. Following energy minimization to resolve steric clashes, the system underwent sequential NVT and NPT equilibration phases with the protein backbone restrained. A 200 ns molecular dynamics (MD) production run was executed under NPT ensemble conditions at 310 K and 1 bar to ensure structural relaxation and conformational sampling. To obtain a representative receptor for docking, cluster analysis was performed on the final 50 ns of the trajectory using the GROMACS gmx cluster tool (Daura et al., 1999).

Molecular docking was subsequently performed using AutoDock Vina (Eberhardt et al., 2021) to assess the binding modes of DABPU-DM (Atglistatin) and its derivatives (DABPU-DE and DABPU-DPh). The docking grid was centered on the catalytic site (Ser47 and Asp66) and encompassed the adjacent hydrophobic cavity. Binding affinities were calculated in kcal·mol^-1^, and the resulting complexes were analyzed via the Protein-Ligand Interaction Profiler (PLIP) (Adasme et al., 2021) to characterize the hydrophobic contacts, van der Waals interactions, and hydrogen bonding networks driving protein-ligand binding pose.

### 2.24 Statistical analysis

All experiments were performed at least three times. Statistical analyses were conducted using GraphPad Prism (v8.4.2; GraphPad Software, La Jolla, CA, USA). Differences among groups were evaluated by one-way analysis of variance (ANOVA) followed by Dunnett’s multiple-comparisons test against the corresponding control group (Dunnett, 1955). Statistical significance was defined as P < 0.05, and significance levels are indicated as P < 0.05, P < 0.01, P < 0.001, and P < 0.0001.

Bliss, ZIP, HSA, and Loewe synergy scores for the combination of DABPU-DE and remdesivir were calculated using SynergyFinder 3.0 as described above, and statistical testing of the synergy scores was performed with the same platform.

Dose–response analyses to determine the half-maximal inhibitory concentration (IC_50_) and half-maximal cytotoxic concentration (CC_50_) were performed in GraphPad Prism (v10.4.1) using a four-parameter logistic (4 PL) nonlinear regression model. Responses were normalized to the vehicle control and baseline-corrected prior to curve fitting; the upper and lower plateaus were unconstrained. IC_50_ and CC_50_ values were estimated by nonlinear regression and are reported with 95% confidence intervals.

## 3 Results

### 3.1 Development of derivatives of DABPU-DM (atglistatin) and a solvent to dissolve them

**FIGURE 1:**
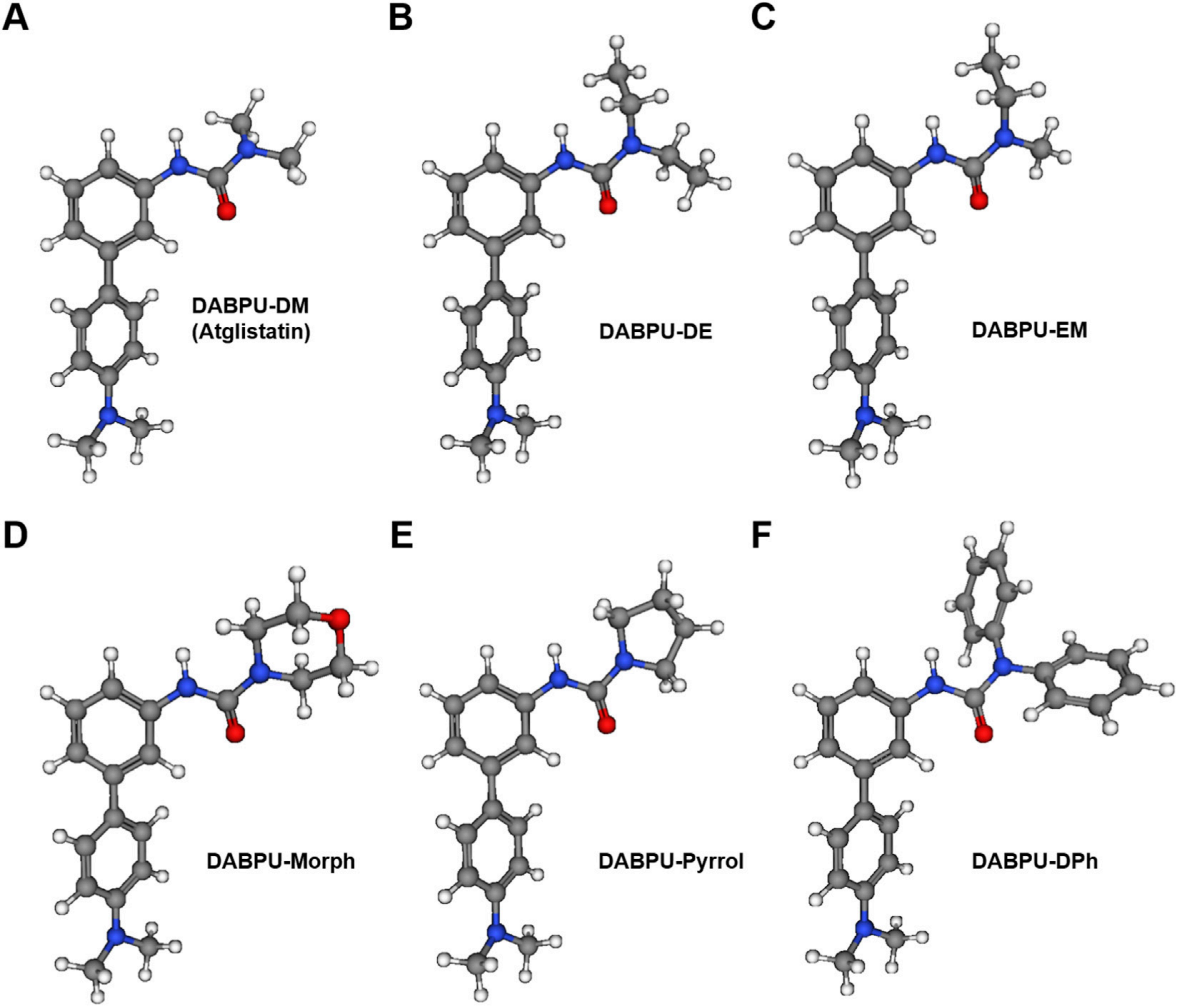
Three-dimensional structure of Atglistatin (DABPU-DM) and its derivatives. **(A)** DABPU-DM. **(B)** DABPU-DE **(C)** DABPU-EM. **(D)** DABPU-Morph **(E)** DABPU-Pyrrol. **(F)** DABPU-DPh. Atglistatin itself consists of 3-(4-(dimethylamino)-[1,1′-biphenyl]-3-yl)-1,1-dimethylurea, while its derivatives include substitutions such as), diethyl (DE), ethylmethyl (EM), morpholine (Morph), pyrrolidine (Pyrrol), or diphenyl (DPh) in place of the terminal dimethylamine group.

**FIGURE 2:**
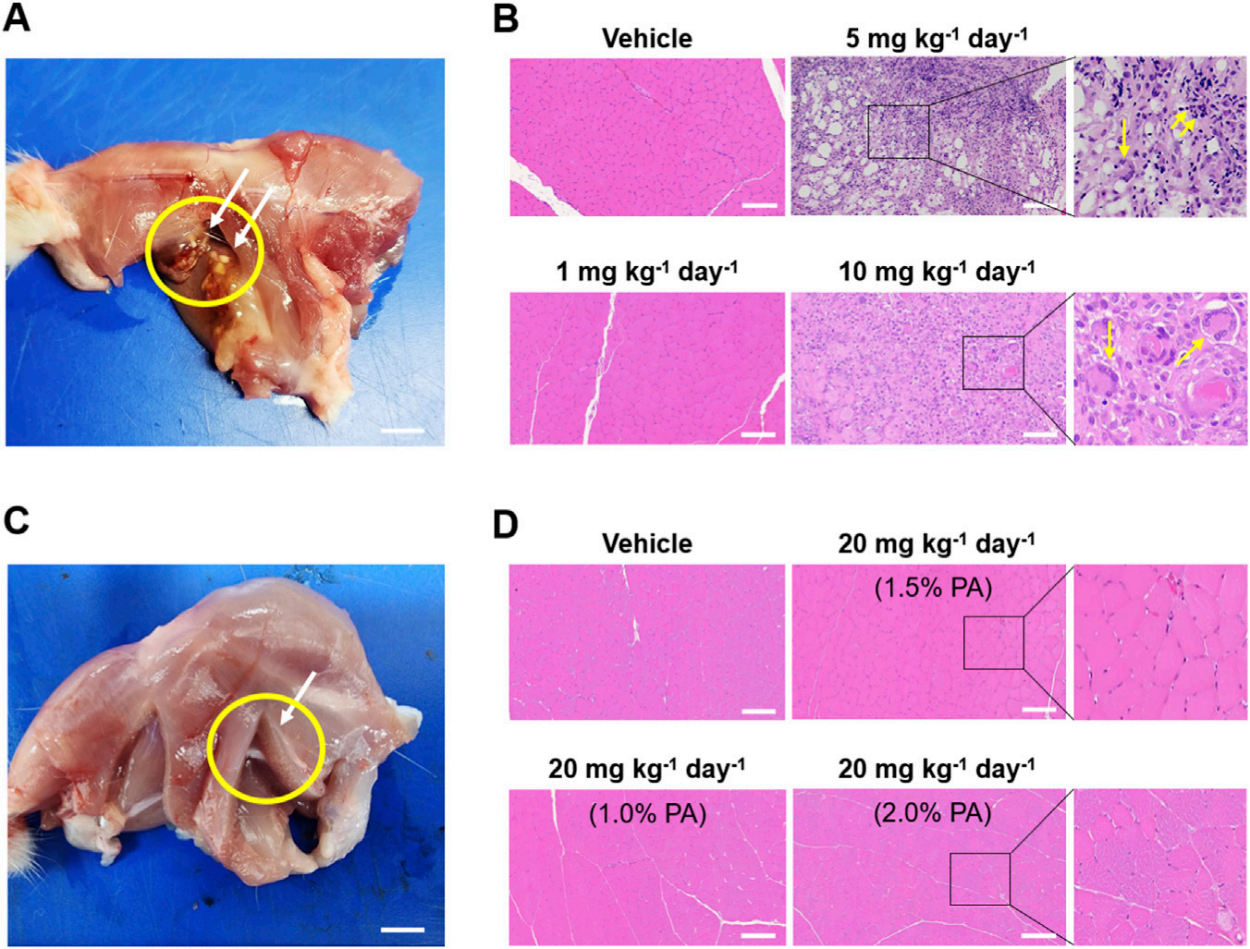
DABPU-DE in DMA-based solvent causes no granulomatous inflammation at the muscle injection site, whereas NMP-based solvent does. DABPU-DE dissolved in NMP- or DMA-based solvents has been injected into the biceps femoris for five consecutive days and then collected from rats euthanized 2 weeks after the first injection. **(A)** Two nodular masses are visible (arrows in the circle) at the muscle injection site with 10 mg kg^−1^ d^−1^ DABPU-DE in NMP-based solvent. **(B)** Granulomatous inflammation is observed at the muscle injection site with 5 and 10 mg kg^−1^ d^−1^ DABPU-DE in NMP-based solvent. These granulomas primarily consist of macrophages, accompanied by Langhans giant cells (arrow) and lymphoid cells (double arrow) (insets). **(C)** Grossly, no significant lesion appears at the muscle injection site (arrow in the circle) with 20 mg kg^−1^ d^−1^ DABPU-DE in DMA-based solvent. **(D)** No histopathological lesions are seen at the muscle injection site with 20 mg kg^−1^ d^−1^ DABPU-DE in different DMA-based solvents with 1.0% phosphoric acid (PA) and 10% phosphate-buffered saline (PBS), 1.5% PA and 10% PBS, and 2.0% PA and 10% PBS (Supplementary Table S5). Muscles in panels B and D are stained with hematoxylin and eosin. Scale bars: A, and C panels = 1 cm; B and D panels = 100 µm.

DABPU-DM (Atglistatin) is known to inhibit multiple RNA viral replications by blocking ATGL, which is essential for the breakdown of LDs during the mid-stage of viral infection. In this study, we developed five derivatives to enhance antiviral efficacy and reduce toxicity (Figure 1). The synthesized derivatives share a common structural framework based on the (4'-(dimethylamino)-[1,1′-biphenyl]-3-yl) urea moiety of DABPU-DM. To ensure the safety, efficacy, and regulatory compliance of DABPU-DM and its derivatives, DABPU-DE, one of its derivatives, was dissolved in NMP- or DMA-based solvents and administered to rats via either intravenous (tail vein) or intramuscular (biceps femoris) routes, respectively. A dose of 20 mg mL^−1^ of DABPU-DE in NMP-based solvent caused precipitation. Therefore, we dissolved DABPU-DE at doses up to 12.5 mg mL^−1^ in NMP-based solvent for subsequent experiments. The highest dose, fully dissolved in the NMP-based solvent, was transparent. Intravenous and intramuscular administration of DABPU-DE (1, 5, and 10 mg kg^−1^ d^−1^) to rats for 5 days in NMP-based solvent did not cause any clinical signs or gross and histopathological changes in the liver, kidneys, heart, lungs, or spleen (data not shown). In contrast, intramuscular dosing at 5 and 10 mg kg^−1^ day^−1^, but not 1 mg kg^−1^ day^−1^, induced multiple whitish nodules at the injection site (Figure 2A). Histologically, these nodules were composed predominantly of macrophages with scattered lymphoid cells and occasional Langhans-type giant cells with a peripheral horseshoe-shaped nuclear arrangement and foreign-body giant cells with randomly distributed nuclei (Figure 2B).

We next dissolved DABPU-DE at doses up to 20 mg mL^−1^ in a DMA-based solvent for subsequent experiments (Supplementary Table S5). The highest concentration of the substance was dissolved in the DMA-based solvent, yielding a transparent solution. In addition, a dose of 20 mg kg^−1^ d^−1^ of DABPU-DE in a DMA-based solvent did not cause any precipitation. Both intravenous and intramuscular administration of rats with DABPU-DE at 20 mg kg^−1^ d^−1^ for 5 days, dissolved in DMA-based solvent, did not induce any clinical signs or gross and histopathological changes in the major organs, including the liver, kidneys, heart, lungs, and spleen (data not shown). Furthermore, intramuscular injections of rats with DABPU-DE at 20 mg kg^−1^ d^−1^ for 5 days, dissolved in a DMA-based solvent, did not cause any gross and histopathological changes at the injection site of the muscles (Figures 2C,D). To adhere to the 3R rule in the use of laboratory animals, we did not perform intramuscular injection experiments at concentrations below 20 mg kg^−1^ d^−1^ of DABPU-DE (Maestri, 2021; Rusche, 2003). These results suggest that DMA-based solvents effectively dissolve Atglistatin and its derivatives, thereby reducing their toxicity. Therefore, in the *in vitro* and *in vivo* experiments below, DABPU-DM and its derivatives were dissolved in DMA-based solvents.

### 3.2 Broad-spectrum safety and antiviral activity of DABPU-DM (atglistatin) and its derivatives *in vitro*

**FIGURE 3:**
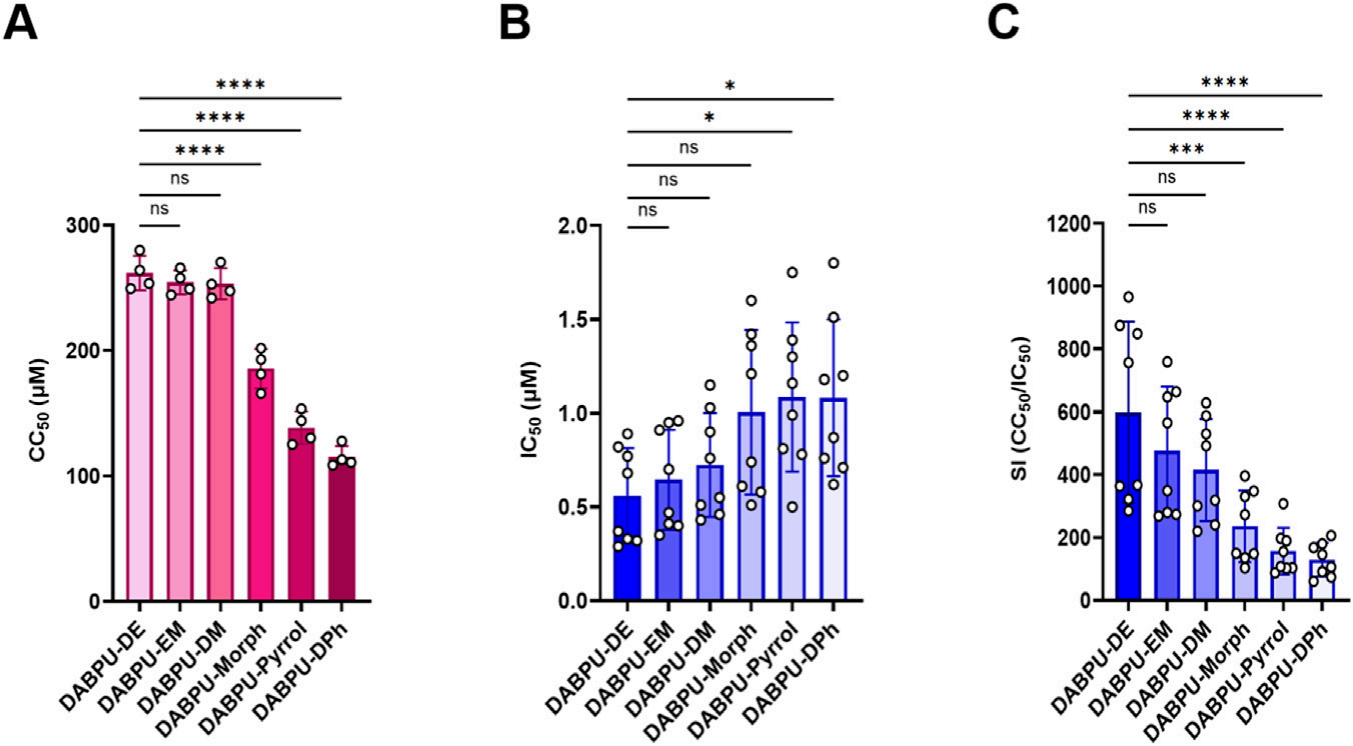
Cytotoxicity and antiviral effects of DABPU-DM (Atglistatin) and its derivatives. **(A)** Cytotoxicity of DABPU-DM and its derivatives was assessed by MTT assay in African green monkey kidney Vero E6, human colorectal adenocarcinoma HRT-18G, chicken embryo kidney (CEK), and swine testicular (ST) cells. The half-maximal cytotoxic concentration (CC_50_) was determined for each compound in each cell line, and mean CC_50_ values from triplicate measurements were plotted. The y-axis indicates compound concentration (µM). **(B)** Antiviral potency of DABPU-DM and its derivatives was evaluated by determining the half-maximal inhibitory concentration (IC_50_) against porcine epidemic diarrhea virus (PEDV), SARS-CoV-2 (KCDC03, lineage A; KDCA51463, Alpha; KDCA55905, Beta; NCCP43426, Omicron BA.5), bovine coronavirus (BCoV), infectious bronchitis virus (IBV), and porcine deltacoronavirus (PDCoV). Mean IC_50_ values from triplicate measurements were plotted. The y-axis indicates compound concentration (µM). **(C)** The selectivity index (SI) was calculated as CC_50_/IC_50_ using the corresponding mean values. Data are presented as mean ± S.D. from three independent experiments. *P < 0.05; **P < 0.01; ***P < 0.001; ****P < 0.0001, using one-way analysis of variance with Dunnett’s correction for multiple comparisons.

**FIGURE 4:**
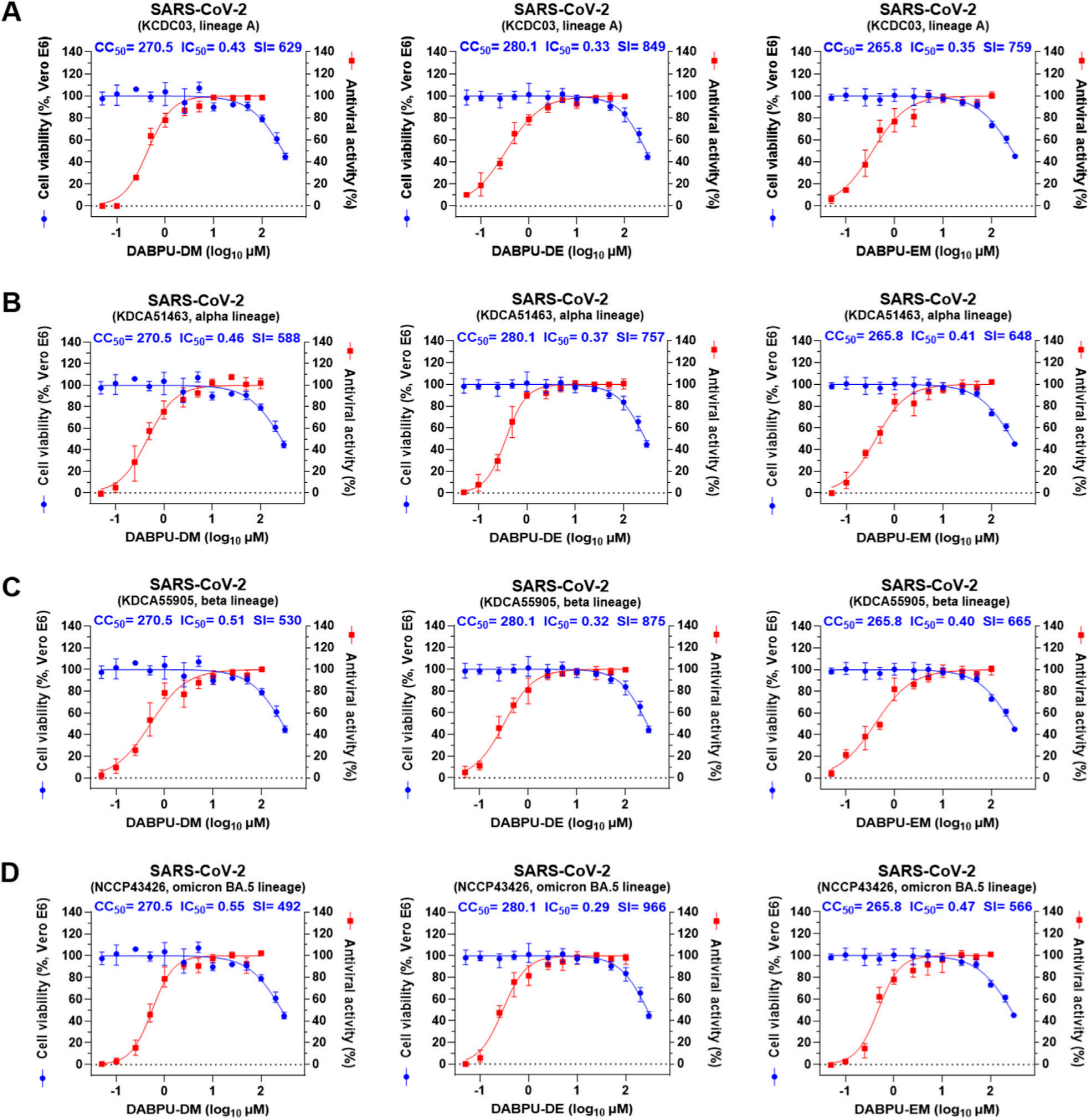
*In vitro* safety and anti-SARS-CoV-2 effectiveness of DABPU-DM (Atglistatin) and its derivatives. The cells infected with each SARS-CoV-2 strain were treated with DABPU-DM (Atglistatin), DABPU-DE, and DABPU-EM at the middle stage of virus replication (18 h post-infection, hpi). **(A–D)** Dose-response curves for half maximal inhibitory concentration (IC_50_), half maximal cytotoxicity concentration (CC_50_), and selectivity index (SI) of DABPU-DM (Atglistatin) and its derivatives against SARS-CoV-2 KCDC03 strain belonging to (A) lineage **(A)**, SARS-CoV-2 KDCA51463 strain in alpha lineage **(B)**, SARS-CoV-2 KDCA55905 strain in beta lineage **(C)**, and SARS-CoV-2 NCCP43426 strain in Omicron BA.5 lineage **(D)**. The viral yield in the cell supernatant was measured using a cell-culture immunofluorescence assay. The cytotoxicity of each small molecule to each cell line was assessed using the MTT assay. Dose-response analyses for IC_50_ and CC_50_ were performed in GraphPad Prism (v10.4.1) using a four-parameter logistic (4 PL) nonlinear regression model. Responses were normalized to the vehicle control and baseline-corrected prior to curve fitting. The upper and lower plateaus were unconstrained, and IC_50_/CC_50_ values were estimated by nonlinear regression with 95% confidence intervals. All data in the graphs are presented as arithmetic means ± S.D. from three independent experiments.

**FIGURE 5:**
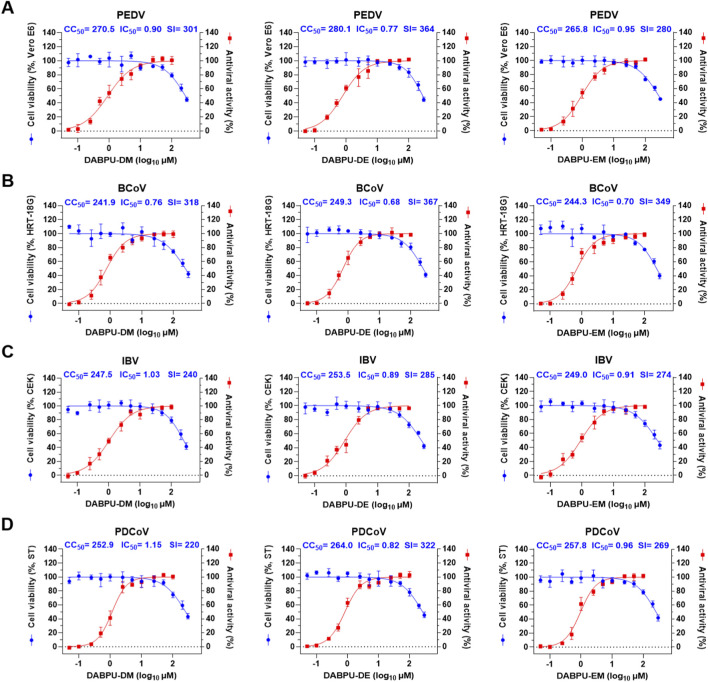
*In vitro* safety and broad-spectrum antiviral effectiveness of DABPU-DM (Atglistatin) and its derivatives. The cells infected with each corresponding virus were treated with DABPU-DM (Atglistatin), DABPU-DE, and DABPU-EM at the middle stage of virus replication: 18 h post-infection (hpi) for porcine epidemic diarrhea virus (PEDV) and bovine coronavirus (BCoV); 24 hpi for infectious bronchitis virus (IBV); and porcine deltacoronavirus (PDCoV). **(A–D)** Dose-response curves for half maximal inhibitory concentration (IC_50_), half maximal cytotoxicity concentration (CC_50_), and selectivity index (SI) of DABPU-DM (Atglistatin) and its derivatives against PEDV QIAP1401 **(A)**, BCoV KWD20 **(B)**, IBV Kr/D49/20 **(C)**, and PDCoV KOR/KNU16-07 strains **(D)**. The viral yield in the cell supernatant was measured using a cell-culture immunofluorescence assay. The cytotoxicity of each small molecule to each cell line was assessed using the MTT assay. Dose-response analyses for IC_50_ and CC_50_ were performed in GraphPad Prism (v10.4.1) using a four-parameter logistic (4 PL) nonlinear regression model. Responses were normalized to the vehicle control and baseline-corrected prior to curve fitting. The upper and lower plateaus were unconstrained, and IC_50_/CC_50_ values were estimated by nonlinear regression with 95% confidence intervals. All data in the graphs are presented as arithmetic means ± S.D. from three independent experiments.

Next, we assessed the safety and antiviral efficacy of DABPU-DM and its derivatives against eight representative viruses from four genera within the subfamily *Orthocoronavirinae* by measuring CC_50_, IC_50_, and SI. As shown in Figure 3, there is a clear trend of safety and antiviral effects of DABPU-DM (Atglistatin) and its derivatives. The cytotoxicity in the cells used for culturing each target virus increased in the order of DABPU-DE, DABPU-EM, DABPU-DM (Atglistatin), DABPU-Morph, DABPU-Pyrrol, and DABPU-DPh (Figure 3A; Supplementary Table S10). To corroborate the cytotoxicity findings, LDH release assays were performed using DABPU-DM (Atglistatin) and its derivatives across multiple cell lines. As shown in Supplementary Figures S1, S2, comparable cytotoxicity profiles were observed in each cell line. Interestingly, antiviral effectiveness against the eight representative coronaviruses showed an inverse relationship, improving in the opposite order (Figure 3B; Supplementary Table S11). This inverse correlation indicates that the highest SI value of DABPU-DE turns it the most favorable for safety and efficacy (Figure 3C; Supplementary Table S12). Notably, DABPU-DE, DABPU-EM, and DABPU-DM (Atglistatin) demonstrated very high safety and antiviral efficacy against both the SARS-CoV-2 prototype and mutant strains (Supplementary Tables S10–S12). These resulted in high SI values, ranging from 492 for the SARS-CoV-2 Omicron strain with DABPU-DM to 966 for the SARS-CoV-2 Omicron strain with DABPU-DE (Figure 4; Supplementary Figure S3). Furthermore, the safety and antiviral effectiveness of DABPU-DE, DABPU-EM, and DABPU-DM (Atglistatin) against SARS-CoV-2 were significantly better than those of representative viruses from other genera within the subfamily *Orthocoronavirinae* (Figure 5; Supplementary Figure S4).

### 3.3 Molecular docking simulation

**FIGURE 6:**
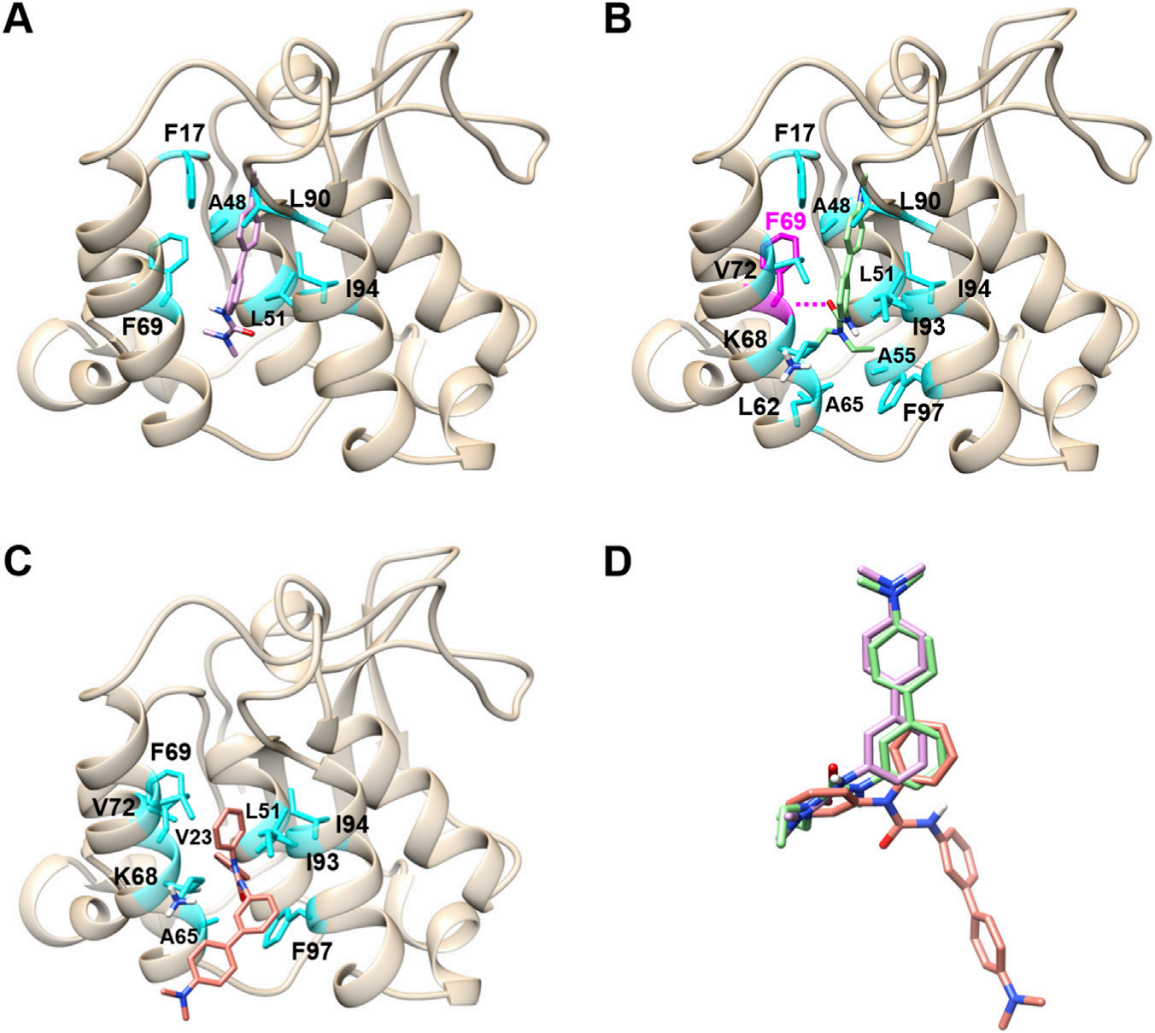
Molecular docking analysis of DABPU-DM (Atglistatin) and its derivatives with human adipose triglyceride (ATGL). Predicted binding modes of **(A)** DABPU-DM (Atglistatin, pink), **(B)** DABPU-DE (green), and **(C)** DABPU-DPh (orange) in the ATGL active site. **(D)** Structural overlay of all three compounds showing different binding orientations. Key hydrophobic interacting residues are shown as cyan sticks, and magenta dashed lines indicate hydrogen bonds.

To investigate the structure-activity relationship (SAR) between the target protein ATGL and its potential inhibitors, we performed molecular docking simulations using the original lead compound, DABPU-DM (Atglistatin), the most potent antiviral derivative, DABPU-DE, and the least potent derivative, DABPU-DPh. The predicted binding energies for DABPU-DM (Atglistatin), DABPU-DE, and DABPU-DPh against human ATGL were −9.4, −9.7, and −8.2 kcal mol^−1^, respectively (Figure 6). The relative differences in binding scores and predicted orientations provide a structural rationale for the variations in observed antiviral activity. DABPU-DM (Atglistatin) was found to occupy the primary hydrophobic cavity of ATGL, primarily through van der Waals and π-alkyl interactions with residues Phe17, Ala48, Leu51, Phe69, Leu90, and Ile94 (Figure 6A). In the case of DABPU-DE, the dimethylurea-to-diethylurea substitution appeared to facilitate deeper penetration into the hydrophobic subpocket compared to the parent compound. This structural refinement enabled the formation of an additional hydrogen bond with the backbone of Phe69 and simultaneously strengthened the hydrophobic network involving Ala55, Leu62, Ala65, Lys68, Val72, Ile93, and Phe97. These more extensive and optimized contacts likely contribute to the slightly enhanced predicted binding affinity (−9.7 kcal⋅mol^−1^) of DABPU-DE and correlate with its superior biological potency (Figure 6B). In contrast, DABPU-DPh, characterized by the incorporation of a bulky diphenylurea moiety, exhibited a markedly different binding behavior. The steric bulk of the diphenyl group appeared to impede the molecule’s ability to fully occupy the hydrophobic cavity that accommodates the dimethylurea group of DABPU-DM (Atglistatin). Consequently, DABPU-DPh adopted a significantly altered orientation toward the cavity entrance. Although it established new interactions with Val23, Ala65, Lys68, Val72, Ile93, and Phe97, it maintained only limited contacts with the core residues Leu51, Phe69, and Ile94 (Figure 6C). This suboptimal binding pose resulted in a substantially reduced predicted affinity (−8.2 kcal⋅mol^−1^). Collectively, these docking simulations suggest that while the diethylurea modification in DABPU-DE optimizes both hydrophobic contacts and hydrogen-bonding motifs to enhance ATGL engagement, the steric hindrance introduced by the diphenylurea group in DABPU-DPh disrupts the conserved binding mode. These findings provide qualitative structural insights into how subtle chemical tuning of the urea moiety can influence the overall efficacy of ATGL-targeted antiviral agents (Figure 6D).

### 3.4 Target engagement and lipolytic activity analyses

To ensure that the superior antiviral potency and *in silico* docking scores of DABPU-DE were not due to indirect cellular responses, we performed a lipase enzymatic activity assay and a cellular thermal shift assay (CETSA) to validate the physical and functional interaction between DABPU-DE and the ATGL enzyme, thereby corroborating our computational predictions with experimental data. The thermal stability of ATGL was evaluated over a temperature range of 40 °C–63 °C. In vehicle-treated controls, ATGL levels declined progressively as temperatures increased, reflective of typical thermal denaturation (Supplementary Figure S5A–C). Conversely, DABPU-DE treatment significantly preserved ATGL protein levels, with marked stabilization observed at 53 °C, 56 °C, and 63 °C (Supplementary Figure S5A–C). These results demonstrate that DABPU-DE confers enhanced thermal resistance to ATGL, providing strong evidence of direct ligand-protein interaction and stabilization. Subsequently, intracellular lipase activity was quantified using a Lipase Assay Kit following treatment with DABPU-DE, CAY10499, a non-specific lipase inhibitor known to inhibit ATGL, hormone-sensitive lipase, and monoacylglycerol lipase (Iglesias et al., 2016). Notably, both lipase inhibitors significantly suppressed total lipase activity compared to the vehicle-treated control (Supplementary Figure S5D). This marked reduction in enzymatic activity could validate functional engagement of ATGL, suggesting the potential of DABPU-DE to modulate viral replication by suppressing LD hydrolysis. Collectively, these target engagement assays provide robust experimental validation that bridges the gap between our *in silico* predictions and biological observations, confirming that the superior antiviral efficacy of DABPU-DE is fundamentally driven by its direct and specific interaction with the ATGL enzyme.

### 3.5 Inhibition of viral replication through blocking ATGL

**FIGURE 7:**
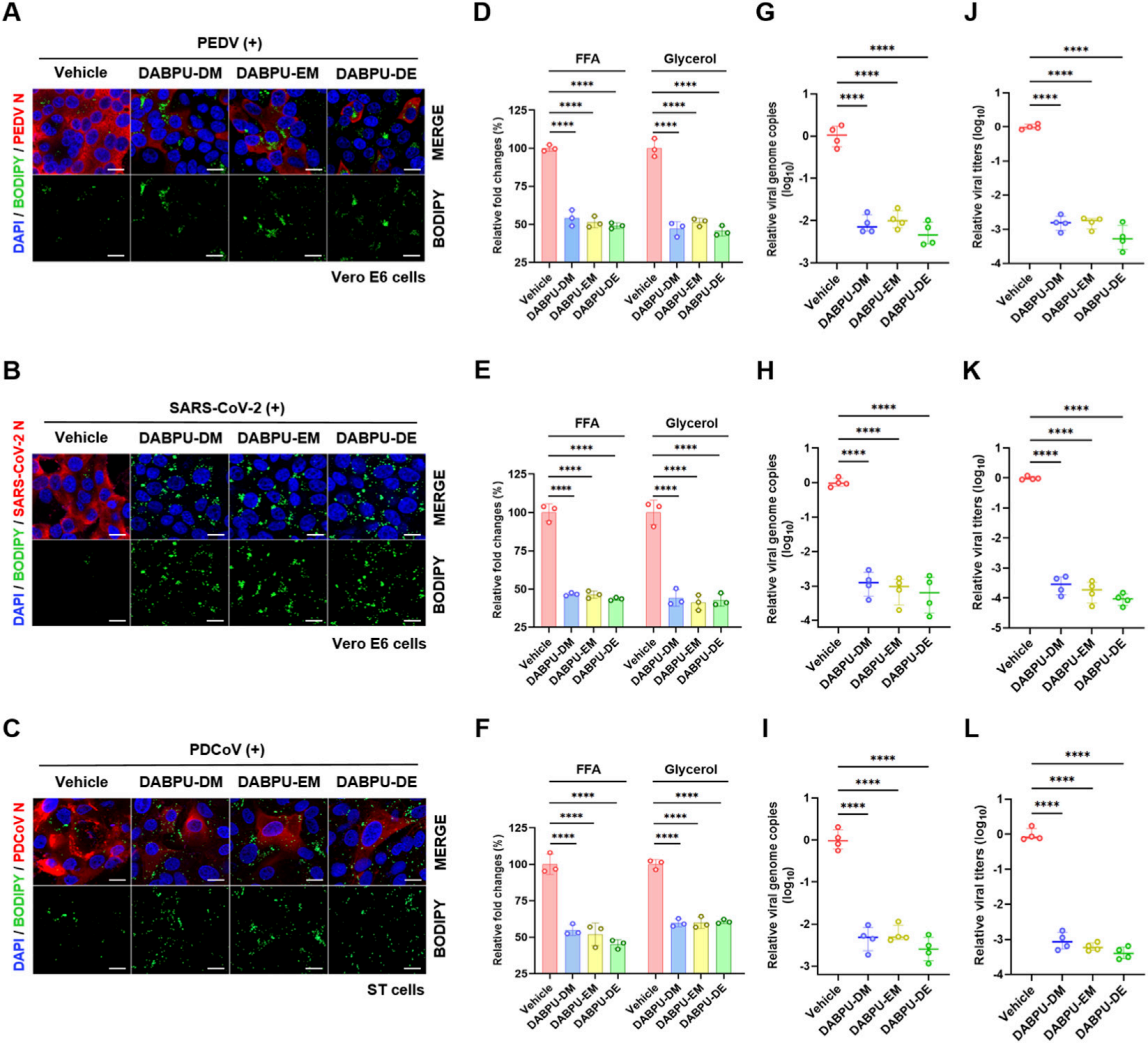
*In vitro* reduction of coronavirus replication through lipolysis inhibition by DABPU-DM (Atglistatin), DABPU-EM, and DABPU-DE. **(A–C)** Retention of BODIPY-positive lipid droplets (LDs, green) with replication inhibition of porcine epidemic diarrhea virus (PEDV) QIAP1401, SARS-CoV-2 KCDC03, and porcine deltacoronavirus (PDCoV) KOR/KNU16-07 strains (red) by treating virus-infected cells (MOI = 0.1 FFU) with 10 µM of each small molecule. These were added at the middle stage of virus replication: 18 h post-infection (hpi) for PEDV and SARS-CoV-2, and 24 hpi for PDCoV. **(D–F)** Inhibition of free fatty acids (FFAs) and glycerol release from LDs following treatment of cells infected with PEDV, SARS-CoV-2, or PDCoV (MOI = 0.1 FFU) with 10 µM of each small molecule. **(G–L)** Reduction in each corresponding viral genome copy number and infectious progeny production after treating cells infected with PEDV, SARS-CoV-2, or PDCoV (MOI = 0.1 FFU) with 10 µM of each small molecule. All data are presented as means ± standard deviation from at least three independent experiments. *P < 0.05; **P < 0.01; ***P < 0.001; ****P < 0.0001, using one-way analysis of variance with Dunnett’s correction for multiple comparisons. Scale bars = 10 µm.

In the experiments above, DABPU-DE, DABPU-EM, and DABPU-DM (Atglistatin) showed superior safety and antiviral effectiveness compared to the other three derivatives. While DABPU-DM (Atglistatin) is a known inhibitor of viral replication that works by blocking ATGL—a lipase that binds to LDs, breaks down TAG, and produces FFAs essential for viral replication—it was still unclear whether DABPU-DE and DABPU-EM share this mechanism. To investigate this possibility, we first examined whether viral replication was impaired in the absence of ATGL. ATGL knockdown reduced viral protein synthesis, genome replication, and viral antigen formation (Supplementary Figures S6A–D). Next, we treated virus-infected cells with 10 µM of DABPU-DE, DABPU-EM, or DABPU-DM during the middle stage of infection. This treatment significantly retained LDs in infected cells, including human lung Calu-3 cells (Figures 7A–C; Supplementary Figures S7A–C). The decrease in LD breakdown was directly linked to a reduction in FFA production (Figures 7D–F; Supplementary Figures S7D,F). As a result, FFA depletion significantly slowed viral replication, including viral protein synthesis (Figures 7A–C; Supplementary Figures S7A–C), viral genome replication (Figures 7G–I; Supplementary Figures S7G–I), and the production of infectious virus particles (Figures 7J–L; Supplementary Figures S7J–L). Interestingly, DABPU-DE also demonstrated potent antiviral efficacy in human lung organoids infected with SARS-CoV-2 (Supplementary Figure S7M). This, combined with the antiviral efficacy observed in Calu-3 cells, suggests that DABPU-DE has high potential for human use. In contrast, supplementation individually with saturated palmitic acid (PA), monounsaturated oleic acid (OA), and polyunsaturated linoleic acid in the above FFA-deprived condition in the cells treated with DABPU-DE significantly rescued SARS-CoV-2 replication (Supplementary Figures S8A,B). Treating infected cells with each compound immediately after virus adsorption resulted in less antiviral activity compared to treatment during the middle of viral replication (Supplementary Figures S9A–D). Overall, these results indicate that DABPU-DE operates via the same mechanism as DABPU-DM (Atglistatin): it inhibits ATGL in infected cells. By blocking the breakdown of LDs and the subsequent production of FFAs, these compounds effectively suppress viral replication. This shared mechanism suggests the therapeutic potential of DABPU-DE, DABPU-EM, and DABPU-DM as host-targeted strategies for combating viral infections.

### 3.6 Antiviral synergy of DABPU-DE with remdesivir

**FIGURE 8:**
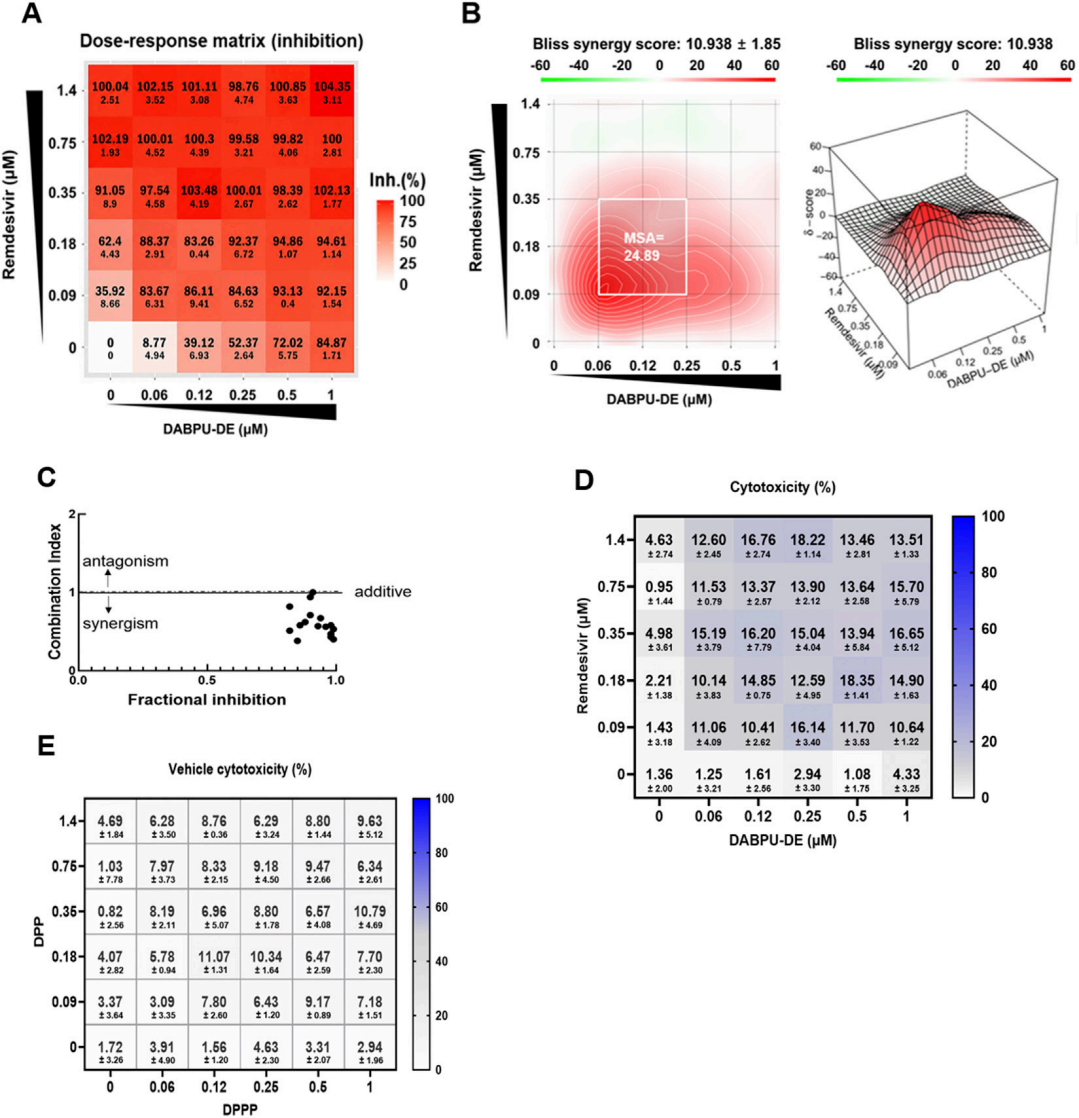
Antiviral synergistic effects and safety of combining DABPU-DE with remdesivir. Vero E6 cells were infected with the SARS-CoV-2 KCDC03 strain (MOI of 0.1) or left uninfected as controls. The cells were treated with specified concentrations of remdesivir immediately after virus absorption and with DABPU-DE at the mid-stage of infection (18 h post-infection), either alone or in combination, then incubated for an additional 18 h. **(A)** Dose-response matrix showing the percent inhibition of infection. **(B)** Two- and three-dimensional landscapes (left and right) display synergy across the dose-response matrix. The Bliss independence reference model was used to determine whether the drug combination acts synergistically. The left landscape highlights the most synergistic area (MSA) within a white box. **(C)** Fractional inhibition (X-axis) versus combination index (Y-axis) plots demonstrate the synergistic effects of DABPU-DE combined with remdesivir. **(D)** Cytotoxicity of DABPU-DE and remdesivir, individually or combined, at different concentrations. **(E)** Cytotoxicity of the vehicles. All data are presented as means ± standard deviation (S.D.) from three independent experiments.

Combined antiviral therapy, which targets different viral and/or host factors, can improve treatment effectiveness while decreasing toxicity, even at lower doses than those used alone (Gibas et al., 2022; Kumar et al., 2020; Marco et al., 2025; Wagoner et al., 2022). Therefore, we assessed the potential synergistic antiviral effects of host ATGL-targeting DABPU-DE combined with SARS-CoV-2 RNA-dependent RNA polymerase (RdRp)-targeting remdesivir. As shown in Figure 8, all drug combinations exhibited dose-dependent synergistic suppression of SARS-CoV-2 infection. Notably, synergistic viral inhibition was achieved at 2- to 5-fold lower concentrations in combination treatments than in monotherapies (Figure 8A). Each drug alone achieved maximum inhibition of 84.87% and 100.04%, respectively. In contrast, the combination achieved 100% inhibition across multiple dose combinations (Figure 8A). The Bliss independence model synergy score is 10.938, indicating a synergistic interaction between the two drugs (Figure 8B). Additionally, the most synergistic area (MSA) score is 24.89, supporting the above results and highlighting significant synergy within a specific concentration range in the 6 × 6 dose matrix (Figure 8B). Consistent results were obtained using additional reference models. The ZIP model produced a synergy score of 11.03 with an MSA score of 24.74, whereas the HSA model generated a synergy score of 15.71 and an MSA score of 32.09, further supporting synergistic antiviral activity. Even the Loewe model indicated an overall additive interaction (synergy score = 4.84), 15.11 of the MSA score supported the above synergistic antiviral activity (Supplementary Figures S10A–C). To further validate these findings, the 6 × 6 dose-response matrix was analyzed using the Chou–Talalay method implemented in Compusyn software. The fraction affected (Fa) and combination index (CI) values for all drug combinations are summarized in (Supplementary Table S13) and illustrated in (Figure 8C). Most combinations exhibited synergism, with CI values ranging from 0.38 to 0.85. Notably, the combination of 0.06 μM DABPU-DE and 0.09 μM remdesivir achieved 85% viral inhibition (Fa = 0.85) with a CI value of 0.38 ± 0.10, indicating synergism according to the Chou–Talalay classification. These findings are consistent with the synergy analyses obtained using the Bliss, ZIP, and HSA models. To assess the safety profile of the combination treatment, cytotoxicity was evaluated in Vero E6 cells following exposure to DABPU-DE, remdesivir, or their combinations. The highest level of cytotoxicity observed was 18.35% when combining 0.5 µM DABPU-DE with 0.18 µM remdesivir at 36 h post-treatment. Importantly, none of the treatment groups exhibited a dose-dependent increase in cytotoxicity, suggesting that the observed antiviral synergy was not associated with enhanced cellular toxicity (Figures 8D,E).

## 4 Discussion

The periodic and sequential emergence of SARS-CoV, MERS-CoV, and SARS-CoV-2 in the early 21st century raises the possibility that SARS-CoV-3, MERS-CoV-2, or another pandemic Coronavirus Disease X may emerge in the near future and threaten human life (Fan et al., 2025; Miranda et al., 2021; Wang et al., 2023). Therefore, the development of a new class of therapeutics capable of targeting a wide range of coronaviruses is essential for pandemic preparedness (Fan et al., 2025; Miranda et al., 2021; Van Damme et al., 2025; Wang et al., 2023). In this study, we evaluated the antiviral efficacy of DABPU-DM (Atglistatin) and five of its derivatives against representative viruses from all four genera (*Alpha-*, *Beta-*, *Gamma-*, and *Deltacoronavirus*) within the subfamily *Orthocoronavirinae*. Our findings suggest that three of these compounds, DABPU-DE, DABPU-EM, and DABPU-DM, exhibit potent antiviral activity at nanomolar concentrations against all tested viruses. Notably, they exhibited excellent antiviral efficacy against not only the SARS-CoV-2 Wuhan-like strain but also against Alpha-, Beta-, and Omicron variants. Furthermore, their potent activity extends to representative viruses from the other coronavirus genera: *Alpha-*, *Gamma-*, and *Deltacoronavirus*. These results suggest that DABPU-DE, DABPU-EM, and DABPU-DM (Atglistatin) hold significant promise as pan-coronavirus therapeutic agents (Fan et al., 2025; Scheriber and Ludwig, 2025; Wang et al., 2023). Their broad-spectrum activity could make them valuable not only for treating current SARS-CoV-2 variants but also for mitigating the impact of future pandemic infections caused by a wide range of coronaviruses (Fan et al., 2025; Kumar et al., 2020; Meganck and Baric, 2021; Scheriber and Ludwig, 2025; Wang et al., 2023).

To further substantiate the mechanistic link between ATGL inhibition and antiviral activity, we validated our findings through genetic approaches. The siRNA-mediated knockdown of ATGL significantly suppressed SARS-CoV-2 replication, effectively recapitulating the antiviral efficacy observed with DABPU-DM and its derivatives. These results align with our current and previous reports, which showed that pharmacological inhibition of ATGL prevents LD degradation in both SARS-CoV-2-infected cells and/or animal models, leading to LD accumulation and a simultaneous decrease in FFA and glycerol levels. Moreover, this causal relationship is further reinforced by current and previous observations that exogenous FFA supplementation rescued viral replication inhibited by DABPU-DM or DABPU-DE. Such metabolic rescue provides compelling evidence that the antiviral effect is fundamentally driven by the modulation of ATGL-mediated lipid substrates rather than by non-specific, off-target interactions.

Although a dual-model system—such as human ACE2-expressing mice on a conditional ATGL-knockout background—was not available for this study to directly demonstrate *in vivo* causality, the physiological importance of ATGL in viral pathogenesis is well supported by the existing literature. Most notably, ATGL-deficient mice exhibit significant resistance to lethal influenza A virus challenges, and DABPU-DM has demonstrated robust *in vivo* efficacy against various SARS-CoV-2 strains. Through this orthogonal validation, our data provide strong evidence that DABPU-DM and its derivatives exert broad-spectrum antiviral activity by specifically targeting ATGL-mediated lipolysis. Our ongoing research aims to further delineate these molecular requirements by utilizing inducible ATGL-knockout mice expressing human ACE2 to characterize the precise temporal role of ATGL throughout the SARS-CoV-2 life cycle.

The effectiveness of therapeutic agents is profoundly influenced by their binding affinities and specific binding modes within the active site of the target protein (Alufi et al., 2022; Mondal et al., 2021). Our computational results suggest a correlation between the predicted docking scores and the observed antiviral potencies of the DABPU derivatives. Specifically, the superior performance of DABPU-DE and DABPU-DM compared to DABPU-DPh may reflect their propensity to form more stable, energetically favorable complexes with ATGL, as suggested by our structural models. However, since computational docking primarily provides theoretical binding modes, it remained to be determined whether the observed antiviral effects of DABPU-DE stemmed from its direct interaction with ATGL or through indirect cellular responses. To address this, we further validated these predictions through functional and biophysical assays. Specifically, we performed a Cellular Thermal Shift Assay (CETSA) to evaluate direct target engagement and a cellular lipase activity assay to assess the functional consequences of compound treatment on cellular lipolytic activity. The CETSA results demonstrated significant thermal stabilization of ATGL following DABPU-DE treatment, providing strong evidence of direct interaction between DABPU-DE and ATGL within the cellular environment. In parallel, the lipase activity assay showed that DABPU-DE significantly reduced total cellular lipase activity, possibly indicating functional suppression of intracellular lipolysis. The observed reduction in lipase activity is consistent with inhibition of ATGL-mediated LD hydrolysis when interpreted together with the CETSA findings. Thus, the combined biophysical and functional data provide complementary experimental support for ATGL as the molecular target of DABPU-DE and indicate that the observed antiviral activity is associated with direct target engagement and suppression of cellular lipolytic activity rather than indirect cellular effects. While the three-dimensional crystal structure of ATGL has not yet been fully elucidated, necessitating a qualitative interpretation of docking-derived binding energies, our integrated approach provides a reliable mechanistic foundation. Efforts to resolve the high-resolution structure of ATGL and conduct co-crystallization studies are currently underway, which will enable a more precise characterization of the inhibitor-protein interface in future investigations (Marković et al., 2024).

In the present study, the sequential treatment of remdesivir and DABPU-DE was strategically designed to target distinct phases of the viral life cycle. This approach is biologically justified by the stage-dependent antiviral efficacy of each compound. DABPU-DE displayed maximal antiviral potency when treated during the mid-infection phase, a period characterized by peak ATGL activity, whereas its efficacy was limited during the early stages. This observation aligns with reports that the inhibitory effect of DABPU-DE against feline infectious peritonitis virus intensifies as treatment timing coincides with peak infection stages (Aboelmagd et al., 2026). Such variations likely stem from the dynamic remodeling of host lipid metabolism and the fluctuating activity of LD-associated proteins, such as ATGL (Papa et al., 2021). These proteins undergo functional shifts throughout the viral life cycle, potentially driven by biophysical changes like liquid-liquid phase separation within the LD environment (Papa et al., 2021). As a result, the optimal therapeutic window for ATGL inhibition appears closely tied to the host’s specific metabolic state during mid-stage viral replication. Conversely, remdesivir was treated immediately following viral adsorption to target the initial stages of viral RNA synthesis. As a nucleoside analog that inhibits the viral RdRp, remdesivir is most effective during the early post-entry phase when viral replication begins (Gordon et al., 2020). Therefore, our sequential protocol—initiating remdesivir treatment at the onset of replication and introducing DABPU-DE at 18 hpi (the peak of host metabolic reorganization)—was designed to simultaneously suppress viral enzyme activity and host-mediated lipid exploitation. This strategic timing maximizes the synergistic potential of the two compounds by targeting the virus at its most vulnerable stages throughout the infection timeline.

In pharmaceutical manufacturing, selecting appropriate solvents is crucial for ensuring the safety, efficacy, and regulatory compliance of a drug product throughout its lifecycle—from synthesis to final formulation (Kolář et al., 2002; Savjani et al., 2012). In a previous study, DABPU-DM (Atglistatin) was dissolved in 10% PEG400 in water (v/v) for intraperitoneal injection into IAV-infected mice and in 50% PEG400 in water (v/v) for intraperitoneal injection into SARS-CoV-2-infected hamsters. However, the resuspended DABPU-DM solution in PEG400 became light milky and caused chronic granulomatous inflammation at the injection site when injected into the muscle of mice or hamsters. To enhance safety, efficacy, and regulatory compliance, DABPU-DE, a derivative of Atglistatin (DABPU-DM), was dissolved in DMA- or NMP-based solvents and administered to rats via tail vein or intramuscular routes. The NMP-based solvent formed a precipitate at a dose of 20 mg mL^−1^, but the DMA-based solvent did not at this dose, indicating that DMA-based solvents dissolve DABPU-DE better (Kolář et al., 2002; Savjani et al., 2012). Both 12.5 mg mL^−1^ of DABPU-DE dissolved in NMP-based solvent and 20 mg mL^−1^ of DABPU-DE dissolved in DMA-based solvent remained transparent. However, 5 mg kg^−1^ d^−1^ and 10 mg kg^−1^ d^−1^ of DABPU-DE in NMP-based solvent caused chronic granulomatous inflammation at the injection site in muscle tissue. Notably, 20 mg kg^−1^ d^−1^ of DABPU-DE in DMA-based solvent did not induce any macroscopic or histopathological changes at the injection site. Our data suggest that DMA-based solvents better solubilize DABPU-DE than PEG400-or NMP-based solvents, making them preferable for improved safety, efficacy, and regulatory compliance (Kolář et al., 2002; Savjani et al., 2012).

In a previous study, DABPU-DM (Atglistatin) was dissolved in DMSO to assess its broad-spectrum *in vitro* antiviral activity. Compared to results with DABPU-DM dissolved in the previous solvent, DABPU-DM dissolved in a DMA-based solvent showed a higher dose for CC_50_ and a lower dose for IC_50_, leading to a better SI in the DMA-based solvent than in DMSO. These findings also indicate that DMA-based solvents dissolve DABPU-DM more safely and effectively (Kolář et al., 2002; Savjani et al., 2012).

The pharmacological relevance of the dosing regimen in the present study warrants careful interpretation, as the primary objective was focused on systemic safety and formulation optimization rather than *in vivo* efficacy testing. This work employed two clinically relevant solvent systems (DMA-based and NMP-based formulations) to characterize the tolerability and potential acute toxicity of DABPU-DM in rats via intravenous (IV) and intramuscular (IM) administration. While the observed safety profile supports the feasibility of DMA-based delivery, it is important to note that these findings do not yet translate to a comprehensive preclinical antiviral safety profile. Specifically, our current data lack detailed assessments of pharmacokinetics, systemic exposure, and bioavailability, which are essential for defining the therapeutic margin and confirming whether efficacious concentrations are achieved in target tissues. Furthermore, as ATGL is a host-directed target with critical physiological roles in lipid homeostasis, the potential for metabolic toxicity and pulmonary tolerability issues stemming from sustained ATGL inhibition remains to be fully elucidated. Consequently, while the absence of acute adverse effects is encouraging, the *in vivo* safety of these compounds as antiviral agents has not yet been established. To address these critical gaps, we are actively pursuing GLP-compliant PK/PD and bioavailability studies as part of an IND-enabling program. These investigations will focus on defining exposure-response relationships, characterizing the metabolic impact of ATGL inhibition in various tissues, and establishing a clear therapeutic window. Such data will be indispensable for guiding subsequent efficacy trials and ensuring the safety of ATGL-targeted therapies in clinical disease models.

**FIGURE 9:**
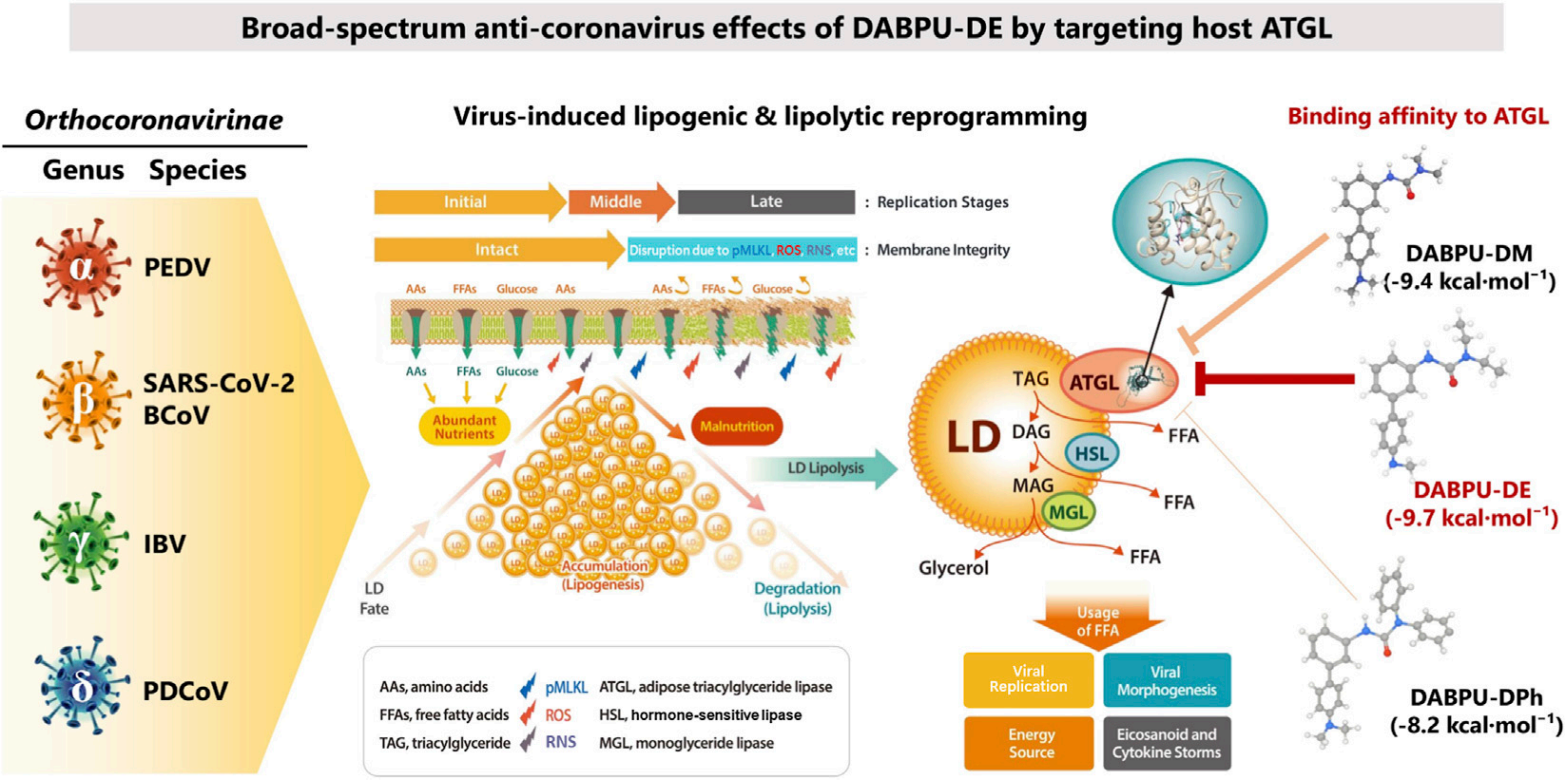
Schematic diagram illustrating the mechanism by which DABPU-DM (Atglistatin) and its derivatives DABPU-DE and DABPU-DPh target host adipose triglyceride lipase (ATGL) to induce broad-spectrum anti-coronavirus effects. Representative viruses from all four genera within the subfamily *Orthocoronavirinae*—porcine epidemic diarrhea virus (PEDV; *Alphacoronavirus*), SARS-CoV-2 (BCoV; *Betacoronavirus*), infectious bronchitis virus (IBV; *Gammacoronavirus*), and porcine deltacoronavirus (PDCoV; *Deltacoronavirus*)—can perturb cellular membranes through virus-induced host factors, including phosphorylated mixed lineage kinase domain-like protein (pMLKL) and reactive oxygen species (ROS). These membrane perturbations contribute to downstream dysfunctions, including altered transporter activity and impaired nutrient uptake. During infection, lipid droplets (LDs) may accumulate, and subsequent lipolysis by host lipases (including ATGL) releases free fatty acids (FFAs) that support late stages of the viral life cycle by promoting viral replication, viral morphogenesis, and energy production, and by contributing to eicosanoid and cytokine synthesis. Among DABPU-DM (Atglistatin) and its five derivatives, DABPU-DE and DABPU-DM showed the highest predicted binding affinities for ATGL (−9.7 and −9.4 kcal⋅mol^−1^, respectively), whereas DABPU-DPh exhibited the lowest affinity (−8.2 kcal⋅mol^−1^). Consistent with these affinities, DABPU-DE and DABPU-DM displayed comparatively stronger antiviral activity, while DABPU-DPh showed reduced efficacy.

Viral infections can perturb cellular membranes, contributing to downstream dysfunctions such as altered transporter activity and impaired nutrient uptake (Figure 9) (Daussy and Wodrich, 2020; Harrison, 2008). In this context, LDs may accumulate during infection, and their subsequent hydrolysis can release FFAs, completing the viral life cycle (Figure 9) (Cesar-Silva et al., 2023). DABPU-DM has been show5n to inhibit viral replication by blocking ATGL, which is essential for the breakdown of accumulated LDs during malnutrition (Cesar-Silva et al., 2023). In this study, DABPU-DE and DABPU-EM, among the DABPU-DM (Atglistatin) derivatives, inhibited LD breakdown in cells infected with various coronaviruses more strongly than DABPU-DM (Figure 9). This caused LDs to remain in infected cells, resulting in lower FFAs and glycerol levels than in the untreated control group. This reduction in FFA production ultimately suppressed replication of representative viruses from four genera within the subfamily *Orthocoronavirinae* (Blázquez et al., 2025; Cesar-Silva et al., 2023; Laufman et al., 2019).

Combination therapy with antiviral agents targeting different mechanisms may reduce toxicity and increase efficacy (Hung et al., 2020). Therapies currently used to treat HIV and HCV also consist of a combination of two or three virus-targeting antiviral drugs (Cihlar and Fordyce, 2016; Sarrazin, 2021). Antiviral agents can either directly target the virus, such as viral proteins or nucleic acids, or target host cell factors involved in viral replication (Kausar et al., 2021). In this study, combining DABPU-DE with remdesivir conferred robust, synergistic suppression of SARS-CoV-2 infection in Vero E6 cells with significantly high synergic and MSA scores. Thus, the DABPU-DE and the RdRp inhibitors may be worth testing in combination (Wagoner et al., 2022).

## 5 Conclusion

In this study, we developed five derivatives of DABPU-DM (Atglistatin), an ATGL inhibitor. DABPU-DE exhibited the strongest antiviral activity against representative viruses from all four genera within the subfamily *Orthocoronavirinae* at nanomolar concentrations (Figure 9). Its superior antiviral efficacy is linked to its diethylurea group, which enhances binding affinity for ATGL through optimized hydrophobic and hydrogen-bonding interactions (Figures 6, 9). Furthermore, the synergistic suppression of SARS-CoV-2 infection observed when combining DABPU-DE with the viral RdRp inhibitor remdesivir suggests its potential utility in combinatorial therapeutic approaches. However, it is important to acknowledge that while these *in vitro* results are promising, the *in vivo* antiviral safety and efficacy of these compounds have yet to be established. Despite this limitation, our findings establish DABPU-DE as a significant early-stage preclinical platform, highlighting the potential of host-directed lipid metabolism targeting as a strategy to address current SARS-CoV-2 variants and enhance preparedness for future coronavirus pandemics.

## Data Availability

The datasets presented in this article are not readily available because the original contributions presented in the study are included in the article/Supplementary Material; further inquiries can be directed to the corresponding authors. Requests to access the datasets should be directed to K-OC, choko@jnu.ac.kr.

## Supplementary material

SUPPLEMENTARY FIGURE S1 Cytotoxicity of DABPU-DM (Atglistatin), DABPU-DE, and DABPU-EM in multiple cell lines. **(A–D)** Cytotoxicity of DABPU-DM (Atglistatin), DABPU-DE, and DABPU-EM was evaluated by LDH release assay in African green monkey kidney Vero E6 **(A)**, human colorectal adenocarcinoma HRT-18G **(B)**, chicken embryo kidney (CEK) **(C)**, and swine testicular (ST) **(D)** cells. Following treatment with the indicated compound concentrations, LDH released into the culture supernatant was quantified using a commercial Cytotoxicity Detection Kit (see Materials and Methods). The x-axis denotes compound concentration (µM), and the y-axis shows cytotoxicity (%) relative to mock-treated cells.

SUPPLEMENTARY FIGURE S2 Cytotoxicity of DABPU-Morph, DABPU-Pyrrol, and DABPU-DPh in multiple cell lines. **(A–D)** Cytotoxicity of DABPU-Morph, DABPU-Pyrrol, and DABPU-DPh was assessed by LDH release assay in African green monkey kidney Vero E6 **(A)**, human colorectal adenocarcinoma HRT-18G **(B)**, chicken embryo kidney (CEK) **(C)**, and swine testicular (ST) **(D)** cells. After treatment with the indicated concentrations of each compound, LDH released into the culture supernatant was quantified using a commercial Cytotoxicity Detection Kit (see Materials and Methods). The x-axis denotes compound concentration (µM), and the y-axis shows cytotoxicity (%) relative to mock-treated cells.

SUPPLEMENTARY FIGURE S3
*In vitro* broad-spectrum safety and anti-SARS-CoV-2 effectiveness of Atglistatin derivatives. DABPU-Morph, DABPU-Pyrrol, and DABPU-DPh were added at the middle stage of virus replication (18 h post-infection, hpi). **(A–D)** Dose-response curves for half maximal inhibitory concentration (IC_50_), half maximal cytotoxicity concentration (CC_50_), and selectivity index (SI) of Atglistatin derivatives against SARS-CoV-2 KCDC03 strain belonging to (A) lineage **(A)**, SARS-CoV-2 KDCA51463 strain in alpha lineage **(B)**, SARS-CoV-2 KDCA55905 strain in beta lineage **(C)**, and SARS-CoV-2 NCCP43426 strain in Omicron BA.5 lineage **(D)**. The viral yield in the cell supernatant was measured using a cell-culture immunofluorescence assay. The cytotoxicity of each small molecule to each cell line was assessed using the MTT assay. Dose-response analyses for IC_50_ and CC_50_ were performed in GraphPad Prism (v10.4.1) using a four-parameter logistic (4 PL) nonlinear regression model. Responses were normalized to the vehicle control and baseline-corrected prior to curve fitting. The upper and lower plateaus were unconstrained, and IC_50_/CC_50_ values were estimated by nonlinear regression with 95% confidence intervals. All data in the graphs are presented as arithmetic means ± S.D. from three independent experiments.

SUPPLEMENTARY FIGURE S4
*In vitro* broad-spectrum safety and anti-viral effectiveness of Atglistatin derivatives. DABPU-Morph, DABPU-Pyrrol, and DABPU-DPh were added at the middle stage of virus replication [18 hours post-infection (hpi) for porcine epidemic diarrhea virus (PEDV) and bovine coronavirus (BCoV); 24 hpi for infectious bronchitis virus (IBV); and porcine deltacoronavirus (PDCoV)]. **(A–D)** Dose-response curves for half maximal inhibitory concentration (IC_50_), half maximal cytotoxicity concentration (CC_50_), and selectivity index (SI) of Atglistatin derivatives against porcine epidemic diarrhea virus (PEDV) QIAP1401 **(A)**, bovine coronavirus (BCoV) KWD20 **(B)**, chicken infectious bronchitis virus (IBV) Kr/D49/20 **(C)**, and porcine deltacoronavirus (PDCoV) KOR/KNU16-07 strains **(D)**. The viral yield in the cell supernatant was measured using a cell-culture immunofluorescence assay. The cytotoxicity of each small molecule to each cell line was assessed using the MTT assay. Dose-response analyses for IC_50_ and CC_50_ were performed in GraphPad Prism (v10.4.1) using a four-parameter logistic (4 PL) nonlinear regression model. Responses were normalized to the vehicle control and baseline-corrected prior to curve fitting. The upper and lower plateaus were unconstrained, and IC_50_/CC_50_ values were estimated by nonlinear regression with 95% confidence intervals. All data in the graphs are presented as arithmetic means ± S.D. from three independent experiments.

SUPPLEMENTARY FIGURE S5 Target engagement and lipolytic activity analyses of DABPU-DE. **(A–C)** Representative Western blot images **(A,B)** and quantification **(C)** of sequential changes of ATGL protein levels following thermal challenge at 40 °C, 46 °C, 49 °C, 53 °C, 56 °C, and 63 °C in vehicle- **(A)** and DABPU-DE-treated **(B)** samples. **(D)** Graphical quantification using a Lipase Assay Kit following treatment with either DABPU-DE or CAY10499, a non-specific lipase inhibitor known to target ATGL, hormone-sensitive lipase, and monoacylglycerol lipase. Relative reduction in total intracellular lipase activity by either DABPU-DE or CAY10499 was compared to the vehicle-treated control. All data in the graphs are presented as arithmetic means ± S.D. from three independent experiments. Differences were evaluated using a one-way ANOVA. **p* < 0.05; ***p* < 0.001; ****p* < 0.0001.

SUPPLEMENTARY FIGURE S6
*In vitro* inhibition of SARS-CoV-2 replication by silencing of adipose triglyceride lipase (ATGL). **(A)** Vero E6 cells were transfected with scrambled siRNA or ATGL-targeting siRNA. ATGL protein levels were quantified by Western blotting and normalized to GAPDH (loading control). **(B–D)** Reduction in SARS-CoV-2 protein **(B)**, genome copy number **(C)**, and intracellular antigen **(D)** in knockdown conditions of ATGL in Vero E6 cells infected with SARS-CoV-2 KCDC03 strain (MOI = 0.1 FFU). All data in the graphs are shown as means ± standard deviation (S.D.) from three independent experiments. *P < 0.05; **P < 0.01; ***P < 0.001; ****P < 0.0001, using one-way analysis of variance with Dunnett’s correction for multiple comparisons.

SUPPLEMENTARY FIGURE S7
*In vitro* suppression of viral replication in cultured cells and human lung organoids by lipolysis inhibition with DABPU-DM (Atglistatin), DABPU-EM, and DABPU-DE. **(A–C)** Representative images showing retention of BODIPY-positive lipid droplets (LDs; green) and reduced viral replication (red) in HRT-18G cells infected with bovine coronavirus (BCoV, MOI = 0.1 FFU), CEK cells infected with infectious bronchitis virus (IBV, MOI = 0.1 FFU), and Calu-3 cells infected with SARS-CoV-2 (MOI = 0.2 FFU) following treatment with 10 µM of the indicated compounds. Compounds were added at the mid-stage of infection (18 hpi for BCoV and SARS-CoV-2; 24 hpi for IBV). **(D–F)** Free fatty acid (FFA) and glycerol release were quantified to assess LD lipolysis in cells infected with BCoV (MOI = 0.1 FFU), IBV (MOI = 0.1 FFU), or SARS-CoV-2 (MOI = 0.2 FFU) and treated with 10 µM of each compound. **(G–L)** Viral genome copy numbers and infectious progeny production were measured in the cells infected with BCoV (MOI = 0.1 FFU), IBV (MOI = 0.1 FFU), or SARS-CoV-2 (MOI = 0.2 FFU), and treated with 10 µM of the indicated compounds. (M) Antiviral activity of DABPU-DE in human lung organoid infected with SARS-CoV-2. Representative confocal images of SARS-CoV-2 nucleocapsid protein. After 14 days of growth in Matrigel with growth factors, lung organoids infected with the SARS-CoV-2 KCDC03 strain at an MOI of 0.5 FFU were vehicle-treated or treated with 10 µM of DABPU-DE at 12 h post-infection (hpi) and incubated further for 12 h. After immunostaining with SARS-CoV-2 nucleoprotein (red), DAPI, and Phalloidin (green), representative three-dimensional images were acquired using a confocal microscope. All data in the graphs are shown as means ± standard deviation (S.D.) from three independent experiments. *P < 0.05; **P < 0.01; ***P < 0.001; ****P < 0.0001, using one-way analysis of variance with Dunnett’s correction for multiple comparisons. Scale bars, 10 µm.

SUPPLEMENTARY FIGURE S8
*In vitro* rescue of SARS-CoV-2 replication inhibited by DABPU-DE treatment through supplementation with free fatty acid (FFA). Vero E6 cells infected with SARS-CoV-2 KCDC03 (MOI = 0.1 FFU) strain were vehicle-treated or treated with 10 µM of DABPU-DE at the middle stage (18 h post-infection, hpi) of infection and then supplemented individually with 100 μM of palmitic acid, oleic acid, or linoleic acid, and incubated further for 18 h **(A,B)** Increase in viral genome copy numbers **(A)** and progeny numbers **(B)** of SARS-CoV-2 with individual supplementation of exogenous palmitic, oleic, or linoleic acids, compared with the vehicle-treated control group. All data in the graphs are shown as means ± standard deviation (S.D.) from three independent experiments. *P < 0.05; **P < 0.01; ***P < 0.001; ****P < 0.0001, using one-way analysis of variance with Dunnett’s correction for multiple comparisons.

SUPPLEMENTARY FIGURE S9
*In vitro* antiviral activities of DABPU-DM (Atglistatin) and its derivatives immediately after absorption of each representative virus. DABPU-DM, DABPU-EM, and DABPU-DE were added immediately after virus absorption. **(A–D)** Reduction in viral genome copy numbers caused by treating cells infected with PEDV **(A)**, SARS-CoV-2 **(B)**, IBV **(C)**, or PDCoV **(D)** at an MOI of 0.1 FFU with 10 µM of each small molecule. All data in the graphs are shown as means ± standard deviation (S.D.) from three independent experiments. *P < 0.05; **P < 0.01; ***P < 0.001; ****P < 0.0001, using one-way analysis of variance with Dunnett’s correction for multiple comparisons.

SUPPLEMENTARY FIGURE S10 Evaluation of antiviral synergistic effects of combining DABPU-DE with remdesivir using multiple drug synergy models. Vero E6 cells were infected with the SARS-CoV-2 KCDC03 strain (MOI of 0.1) or left uninfected as controls. The cells were treated with specified concentrations of remdesivir immediately after virus absorption and with DABPU-DE at the mid-stage of infection (18 h post-infection), either alone or in combination, then incubated for an additional 18 h. **(A–C)** The combinatorial effects of DABPU-DE and remdesivir were assessed using the Zero Interaction Potency (ZIP) **(A)**, the Highest Single Agent (HSA) **(B)**, and the Loewe additivity models **(C)**. Two-dimensional synergy heatmaps (upper panels) and three-dimensional synergy landscapes (lower panels) illustrate the distribution of interaction effects across the tested concentration matrix.

## Glossary

ACC: Acetyl-CoA Carboxylase
IFA: Immunofluorescence assay
AF: Alexa Fluor
IgG: Immunoglobulin G
α-MEM: alpha Minimum Essential Medium
LD: Lipid droplet
BCoV: Bovine coronavirus
Mab: Monoclonal antibody
**CC**_**50**_: half-maximal cytotoxic concentration
MERS: Middle East respiratory syndrome
CCIF: Cell culture immunofluorescence
MTT: 3-(4,5-dimethylthiazol-2-yl)-2,5-diphenyl tetrazolium bromide
CEK: Chicken embryo kidney
N: Nucleocapsid
CI: Confidence interval
NMP: N-methyl-2-pyrrolidone
DMSO: Dimethyl sulfoxide
OD: Optical density
ELISA: Enzyme-linked immunosorbent assay
Pab: Polyclonal antibody
Fa: Fractional anisotropy
PBS: Phosphate-buffered saline
FAS: Fatty Acid Synthase
PBS-NCS: PBS (pH 7.4) containing 0.1% newborn calf serum
DABPU-DE: 3-(4'-(dimethylamino)-[1,1′-biphenyl]-3-yl)) urea-diethyl
PFA: Paraformaldehyde
DABPU-DM: 3-(4'-(dimethylamino)-[1,1′-biphenyl]-3-yl)) urea-dimethyl
PI: Propidium iodide
DABPU-EM: 3-(4'-(dimethylamino)-[1,1′-biphenyl]-3-yl)) urea-ethylmethy
PDCoV: Porcine deltacoronavirus
PEDV: porcine epidemic diarrhea virus
DABPU-DPh: 3-(4'-(dimethylamino)-[1,1′-biphenyl]-3-yl)) urea-diphenyl
RdRp: RNA-dependent RNA polymerase
DABPU-Morph: 3-(4'-(dimethylamino)-[1,1′-biphenyl]-3-yl)) urea-morph
SARS-CoV-2: Severe acute respiratory syndrome coronavirus 2
DABPU-Pyrrol: 3-(4'-(dimethylamino)-[1,1′-biphenyl]-3-yl)) urea-pyrrol
DMA, N: N-dimethylacetamide
SI: Selectivity Index
DMEM: Dulbecco’s Modified Eagle’s Medium
SREBPs: Sterol regulatory element-binding proteins
DPBS: Dulbecco’s phosphate-buffered saline
OA: Oleic acid
FBS: Fetal bovine serum
PA: Palmitic acid
ST: Swine testicular
LA: Linoleic acid
**IC**_**50**_: Half-maximal inhibitory concentration
