# Supplementary material for: DABPU-DE targeting host adipose triglyceride lipase provides broad-spectrum anti-coronavirus effects in vitro
Source: Front Pharmacol. 2026 Jul 29;17:1786048. doi: 10.3389/fphar.2026.1786048 (PMC13473920; doi:10.3389/fphar.2026.1786048)
Supplement: Supplementary file 1 [file Supplementaryfile1.pdf]

# Supplementary Tables

**Supplementary Table S1. Information on cell lines used in this study**

| Cell line | Origin | Source | Medium | Supplementation |
|---|---|---|---|---|
| Vero E6 | African green monkey kidney epithelium | ATCC | DMEM | 10% FBS, 1% P/S |
| Calu-3 | Human lung adenocarcinoma | ATCC | DMEM | 10% FBS, 1% P/S |
| HRT-18G | Human colorectal adenocarcinoma | ATCC | DMEM | 10% FBS, 1% P/S |
| ST | Swine testicular | ATCC | α-MEM | 10% FBS, 1% P/S |
| CEK | Chicken embryo kidney | Prepared | DMEM | 10% FBS, 1% P/S |

Abbreviations: ATCC, American Type Culture Collection; FBS, fetal bovine serum; DMEM, Dulbecco's modified Eagle's medium; α-MEM, Minimal Essential Medium alpha modification; 1% P/S, 100 U/mL penicillin and 100 μg/mL streptomycin.

**Supplementary Table S2. Information on lung organoids used in this study**

| Organoid | Origin | Source | Medium | Supplementation |
|---|---|---|---|---|
| Lung organoid | Human lung adenocarcinoma | KCLB | Lung organoid media | AdDMEM/F12, Antibiotic-antimycotic, GlutaMAX supplement, HEPES, B-27 supplement, R-Spondin, Noggin, FGF-10, FGF-7, N-Acetylcysteine, Nicotinamide, SB202190 monohydrochloride hydrate, A83-01Y-27632 dihydrochloride |

Abbreviations: KCLB, Korean Cell Line Bank; AdDMEM/F12, advanced Dulbecco's Modified Eagle's Medium/F12; HEPES, hydroxyethyl piperazine Ethane Sulfonic acid; FGF-10, fibroblast growth factor-10; FGF-7, fibroblast growth factor-7.

**Supplementary Table S3. Information of virus strains used in this study**

| Genus | Viruses | Strain name (genotypes) | Source |
|---|---|---|---|
| *Alphacoronavirus* | Porcine epidemic diarrhea coronavirus (PEDV) | QIAP1401 (G2b) | Animal and Plant Quarantine Agency, Korea |
| *Betacoronavirus* | Severe acute respiratory syndrome coronavirus 2 (SARS-CoV-2) | KCDC03 (Lineage A) | Korea Disease Control and Prevention Agency |
| | | KDCA51463 (Alpha lineage) | Korea Disease Control and Prevention Agency |
| | | KDCA55905 (Beta lineage) | Korea Disease Control and Prevention Agency |
| | | NCCP43426 (Omicron BA.5 lineage) | Korea Disease Control and Prevention Agency |
| *Betacoronavirus* | Bovine coronavirus (BCoV) | KWD20 | Isolated from fecal samples and propagated in HRT-18G cells |
| *Gammacoronavirus* | Infectious bronchitis virus (IBV) | Kr/D49/20 | Animal and Plant Quarantine Agency, Korea |
| *Deltacoronavirus* | Porcine deltacoronavirus (PDCoV) | KOR/KNU16-07 | Animal and Plant Quarantine Agency, Korea |

**Supplementary Table S4. Information for the culture of porcine epidemic diarrhea virus (PEDV), severe acute respiratory syndrome coronavirus 2 (SARS-CoV-2), bovine coronavirus (BCoV), infectious bronchitis virus (IBV), and porcine detacoronavirus (PDCoV)**

| Viruses | Cell lines | Medium | Supplementation |
|---|---|---|---|
| PEDV | Vero E6 | DMEM | 3 μg/mL porcine pancreatic trypsin, 1% P/S |
| SARS-CoV-2 | Vero E6 / Calu-3 | DMEM | 1 μg/mL TPCK-treated trypsin, 1% P/S |
| BCoV | HRT-18G | DMEM | 5 μg/mL porcine pancreatin, 1% P/S |
| IBV | CEK | DMEM | 1 μg/mL TPCK-treated trypsin, 1% P/S |
| PDCoV | ST | α-MEM | 1 μg/mL porcine pancreatin, 1% P/S |

Abbreviations: CEK, chicken embryo kidney cells; ST, swine testicular cells; DMEM, Dulbecco's modified Eagle's medium; α-MEM, alpha Minimal Essential Medium; 1% P/S: 100 U/mL penicillin, 100 μg/mL streptomycin; TPCK-treated trypsin, N-tosyl-L-phenylalanine chloromethyl ketone (TPCK)-treated trypsin; HEPES, 4-(2-hydroxyethyl)-1-piperazineethanesulfonic acid.

**Supplementary Table S5. Composition of the DMA-based solvent used to dissolve DABPU-DE**

| Group | DMA[1] | PEG400[2] | PA[3] | PBS[4] | Dose (DAPUB-DE) |
|---|---|---|---|---|---|
| 1 | 20% | 60% | 2.0% | 10.0% | None |
| 2 | 20% | 60% | 1.0% | 10.0% | 20 mg $kg^{-1}$ $d^{-1}$ |
| 3 | 20% | 60% | 1.5% | 10.0% | 20 mg $kg^{-1}$ $d^{-1}$ |
| 4 | 20% | 60% | 2.0% | 10.0% | 20 mg $kg^{-1}$ $d^{-1}$ |

[1]DMA; N, N-dimethylacetamide. [2]PEG400, polyethylene glycol 400. [3]PA, phosphoric acid. [4]PBS, phosphate-buffered saline.

## Supplementary Table S6. Chemicals and kits used in this study

| Regents | Company | City/State | Country | Solvent[1] |
|---|---|---|---|---|
| **Chemicals** | | | | |
| Matrigel | Sigma Aldrich | St. Louis, MO | USA | - |
| Atglistatin (DABPU-DM) | MedChem Express | Monmouth Junction, NJ | USA | DMA |
| DABPU-DE | In this study | - | - | DMA |
| DABPU-EM | In this study | - | - | DMA |
| DABPU-DPh | In this study | - | - | DMA |
| DABPU-Pyrrol | In this study | - | - | DMA |
| DABPU-Morph | In this study | - | - | DMA |
| CAY10499 | Cayman Chemicals | Ann Arbor, MI | USA | DMA |
| Remdesivir | Sigma Aldrich | St. Louis, MO | USA | DMA |
| Bodipy 493/503 | Sigma Aldrich | St. Louis, MO | USA | PBS |
| MTT | Sigma Aldrich | St. Louis, MO | USA | PBS |
| Triton X-100 | Sigma Aldrich | St. Louis, MO | USA | PBS |
| Slow-Fade Gold antifade reagent | Molecular Probes | Eugene, OR | USA | - |
| Paraformaldehyde | Sigma Aldrich | St. Louis, MO | USA | - |
| Palmitic acid | Sigma Aldrich | St. Louis, MO | USA | PBS |
| Oleic acid | Sigma Aldrich | St. Louis, MO | USA | PBS |
| Linoleic acid | Sigma Aldrich | St. Louis, MO | USA | PBS |
| AF488 Phalloidin | Thermo Scientific | Waltham, MA | USA | - |
| **Kits** | | | | |
| RNeasy mini kit | Qiagen | Hilden | Germany | - |
| BCA protein assay kit | Thermo Scientific | Waltham, MA | USA | |
| Free fatty acid quantification kit | Abcam | Cambridge, MA | USA | - |
| Free glycerol quantification kit | Abcam | Cambridge, MA | USA | |
| SensiFAST SYBER Lo-ROX one-Step kit | Meridian BIOSCIENCE | Cincinnati, OH | USA | |
| LDH assay kit | Abcam | Cambridge, MA | USA | |
| Lipase assay kit | Sigma Aldrich | St. Louis, MO | USA | |
| **siRNAs** | | | | |
| ON-TARGETplus siRNAs against ATGL (set of 3) | Bioneer Inc | Daejeon | South Korea | RNAse-free water |
| Lipofectamine3000 | Thermo Scientific | Waltham, MA | USA | - |

[1]DMA, N, N-Dimethylacetamide (DMA)-based solvent, consisting of 20% (v/v) DMA, 60% (v/v) polyethylene glycol 400, 2% phosphoric acid, and PBS (pH 7.4).

**Supplementary Table S7. Antibodies used in this study**

| Antibodies[1] | Host | Type[2] | Conjugation | Company | City/State | Country | Solvent[3] |
|---|---|---|---|---|---|---|---|
| **Primary antibodies** | | | | | | | |
| PEDV N | Mouse | Mab | - | Medgene Labs | Brookings, SD | USA | TBST (5% BSA) |
| SARS-CoV-2 S | Rabbit | Pab | - | abcam | Cambridge | UK | TBST (5% BSA) |
| SARS-CoV-2 N | Mouse | Mab | - | Cell Signaling | Beverly, MA | USA | TBST (5% BSA) |
| BCoV N | Mouse | Mab | - | Abcam | Cambridge | UK | TBST (5% BSA) |
| IBV N | Mouse | Mab | - | Novus | Centennial, CO | USA | TBST (5% BSA) |
| PDCoV N | Mouse | Pab | - | Humimmu | Salem, NH | USA | TBST (5% BSA) |
| ATGL | Rabbit | Pab | - | Cell Signaling | Beverly, MA | USA | TBST (5% BSA) |
| GAPDH | Mouse | Mab | | Santa Cruz | Dallas, TX | USA | TBST (5% BSA) |
| **Secondary antibodies** | | | | | | | |
| AF594 rabbit IgG | Donkey | Pab | AF594 | Thermo Scientific | Waltham, MA | USA | 1xPBS |
| AF488 rabbit IgG | Donkey | Pab | AF488 | Thermo Scientific | Waltham, MA | USA | 1xPBS |
| AF594 mouse IgG | Goat | Pab | AF594 | Thermo Scientific | Waltham, MA | USA | 1xPBS |
| AF488 mouse IgG | Goat | Pab | AF488 | Thermo Scientific | Waltham, MA | USA | 1xPBS |
| Rabbit IgG(H+L) | Goat | Pab | HRP | Cell Signaling | Beverly, MA | USA | 1xTBST |
| Mouse IgGκ | Goat | Pab | HRP | Santa Cruz | Dallas, TX | USA | 1xTBST |

[1]Abbreviation: PEDV N, PEDV nucleoprotein; SARS-CoV-2 S, SARS-CoV-2 spike; SARS-CoV-2 N, SARS-CoV-2 nucleocapsid; BCoV N, BCoV nucleocapsid protein; IBV N, IBV nucleocapsid; PDCoV N, PDCoV nucleocapsid; and ATGL, Adipose triglyceride lipase. [2]Mab or Pab, monoclonal or polyclonal antibodies; and HRP, horseradish peroxidase. [3]1xTBST, 1x Tris-Buffered Saline containing 0.1% Tween® 20 Detergent; TBST (5% BSA), 1x TBST containing 5% bovine serum albumin.

**Supplementary Table S8. Experimental design for determining *in vitro* broad-spectrum antiviral effect of Atglistatin and its derivatives at different treatment times**

| Viruses[a] | Cell line | MOI | Drug concentration | Treatment at[b] | Incubation time | Treatment at[c] | Further incubation time[d] | Determination of virus replication |
|---|---|---|---|---|---|---|---|---|
| PEDV | Vero E6 | 0.1 FFU | - | - | 36 hpi | - | 36 hpi | IFA, CCIF, and RT-qPCR |
| | | 0.1 FFU | 10 μM | AVA | 36 hpi | 18 hpi | 18 hpi | IFA, CCIF, and RT-qPCR |
| SARS-CoV-2 | Vero E6 | 0.1 FFU | - | - | 36 hpi | - | 36 hpi | IFA, CCIF, and RT-qPCR |
| | | 0.1 FFU | 10 μM | AVA | 36 hpi | 18 hpi | 18 hpi | IFA, CCIF, and RT-qPCR |
| | Calu-3 | 0.2 FFU | - | - | - | - | 36 hpi | IFA, CCIF, and RT-qPCR |
| | | 0.2 FFU | 10 μM | - | - | 18 hpi | 18 hpi | IFA, CCIF, and RT-qPCR |
| | HLOs | 0.5 FFU | - | - | - | - | 24 hpi | IFA |
| | | 0.5 FFU | 10 μM | - | - | 12 hpi | 12 hpi | IFA |
| BCoV | HRT-18G | 0.1 FFU | - | - | - | - | 36 hpi | IFA, CCIF, and RT-qPCR |
| | | 0.1 FFU | 10 μM | - | - | 18 hpi | 18 hpi | IFA, CCIF, and RT-qPCR |
| IBV | CEK | 0.1 FFU | - | - | 48 hpi | - | 48 hpi | IFA, CCIF, and RT-qPCR |
| | | 0.1 FFU | 10 μM | AVA | 48 hpi | 24 hpi | 24 hpi | IFA, CCIF, and RT-qPCR |
| PDCoV | ST | 0.1 FFU | - | - | 48 hpi | - | 48 hpi | IFA, CCIF, and RT-qPCR |
| | | 0.1 FFU | 10 μM | AVA | 48 hpi | 24 hpi | 24 hpi | IFA, CCIF, and RT-qPCR |

[a]Abbreviations: PEDV, porcine epidemic diarrhea coronavirus; SARS-CoV-2, severe acute respiratory syndrome coronavirus 2; BCoV, bovine coronavirus; IBV, infectious bronchitis virus 4; PDCoV, porcine deltacoronavirus; HLOs, human lung organoids; MOI, multiplicity of infection; FFU, fluorescence focus unit; RT-qPCR, reverse transcription quantitative polymerase chain reaction; CCIF, cell culture immunofluorescence assay.

[b]After virus absorption (AVA).

[c]Hour post-infection.

[d]Incubation time after treatment with the drug.

**Supplementary Table S9. Primers used for the detection of viral and host genes**

| Name[a] | Sequence | Size of amplicon | References |
|---|---|---|---|
| SARS-CoV-2 N | F: 5’-TAATCAGACAAGGAACTGATTA-3’ | 109 | Ogando et al., 2020 |
| | R: 5’-CGAAGGTGTGACTTCCATG -3’ | | |
| PEDV N | F: 5’-GCTATGCTCAGATCGCCAGT-3’ | 92 | Kim et al., 2017 |
| | R: 5’-TCTCGTAAGAGTCCGCTAGCTC-3’ | | |
| BCoV N | F: 5’-TGGATCAAGATTAGAGTTGGC-3’ | 236 | Amer & Almajhdi, 2011 |
| | R: 5’-CCTTGTCCATTCTTCTGACC-3’ | | |
| IBV N | F: 5’-GACGGAGGACCTGATGGTAA-3’ | 206 | Farooq et al., 2024 |
| | R: 5’- CCCTTCTTCTGCTGATCCTG-3’ | | |
| PDCoV M | F: 5’-ATCGACCACATGGCTCCAA-3’ | 72 | Wang et al., 2018 |
| | R: 5’-CAGCTCTTGCCCATGTAGCTT-3’ | | |
| Human GAPDH | F: 5’-ATTCCACCCATGGCAAATTC-3’ | 90 | Baek et al., 2022 |
| | R: 5’-CGCTCCTGGAAGATGGTGAT-3’ | | |
| Porcine GAPDH | F: 5’-ACCTCCACTACATGGTCTACA-3’ | 90 | Deng et al., 2021 |
| | R: 5’-ATGACAAGCTTCCCGTTCTC-3’ | | |
| Chicken GAPDH | F: 5’-TGCTGCCCAGAACATCATCC-3’ | 142 | Truong et al., 2016 |
| | R: 5’-ACGGCAGGTCAGGTCAACAA-3’ | | |

*Abbreviations: SARS-CoV-2 N, severe acute respiratory syndrome coronavirus 2 nucleocapsid protein gene; PEDV N, porcine epidemic diarrhea coronavirus nucleoprotein gene; BCoV N, bovine coronavirus nucleocapsid protein gene; IBV N, infectious bronchitis nucleocapsid protein gene; PDCoV M, porcine deltacoronavirus membrane protein gene; Human GAPDH, human glyceraldehyde 3-phosphate dehydrogenase gene; Porcine GAPDH, porcine glyceraldehyde 3-phosphate dehydrogenase gene; Chicken GAPDH, chicken glyceraldehyde 3-phosphate dehydrogenase gene.

**Supplementary Table S10. The median cytotoxic concentration ($CC_{50}$) of DABPU-DM (Atglistatin) and its derivatives in different cell lines was determined by measuring the activity of the cellular NAD (P) H-dependent cellular oxidoreductase enzymes (MTT assay)**

| Cell line | $CC_{50}$ | | | | | |
|---|---|---|---|---|---|---|
| | DABPU-DE | DABPU-EM | DABPU-DM (Atglistatin) | DABPU-Morph | DABPU-Pyrrol | DABPU-DPh |
| Vero E6 | 280.1 ± 2.03 | 265.8 ± 4.39 | 270.5 ± 3.84 | 201.9 ± 5.34 | 153.8 ± 3.96 | 127.8 ± 2.90 |
| HRT-18G | 249.3 ± 1.90 | 244.3 ± 2.56 | 241.9 ± 2.36 | 181.3 ± 1.93 | 144.3 ± 3.10 | 108.7 ± 3.56 |
| CEK | 253.5 ± 7.17 | 249.0 ± 3.07 | 247.5 ± 2.93 | 165.9 ± 2.41 | 130.3 ± 2.40 | 110.8 ± 4.62 |
| ST | 264.0 ± 3.07 | 257.8 ± 3.01 | 252.9 ± 3.00 | 192.9 ± 1.99 | 125.2 ± 1.51 | 113.0 ± 3.45 |

**Supplementary Table S11. The half maximal (50%) inhibitory concentration ($IC_{50}$) of Atglistatin and its derivatives for coronaviruses**

| Genus | Virus (strain)[1] | $IC_{50}$ | | | | | |
|---|---|---|---|---|---|---|---|
| | | **DABPU-DE** | **DABPU-EM** | **DABPU-DM (Atglistatin)** | **DABPU-Morph** | **DABPU-Pyrrol** | **DABPU-DPh** |
| *Alphacoronavirus* | PEDV (QIAP1401) | 0.77 ± 0.06 | 0.95 ± 0.05 | 0.90 ± 0.07 | 1.36 ± 0.09 | 1.75 ± 0.1 | 1.20 ± 0.06 |
| *Betacoronavirus* | SARS-CoV-2 (KCDC03) | 0.33 ± 0.02 | 0.35 ± 0.03 | 0.43 ± 0.03 | 0.51 ± 0.05 | 0.78 ± 0.08 | 0.87 ± 0.07 |
| | SARS-CoV-2 (KDCA51463) | 0.37 ± 0.02 | 0.41 ± 0.03 | 0.46 ± 0.04 | 0.61 ± 0.05 | 0.81 ± 0.07 | 0.71 ± 0.06 |
| | SARS-CoV-2 (KDCA55905) | 0.32 ± 0.02 | 0.40 ± 0.03 | 0.51 ± 0.05 | 0.58 ± 0.04 | 0.50 ± 0.02 | 0.62 ± 0.03 |
| | SARS-CoV-2 (Omicron, BA.5) | 0.29 ± 0.02 | 0.47 ± 0.03 | 0.55 ± 0.03 | 0.74 ± 0.04 | 0.99 ± 0.1 | 0.76 ± 0.05 |
| *Betacoronavirus* | BCoV (KWD20) | 0.68 ± 0.03 | 0.70 ± 0.05 | 0.76 ± 0.03 | 1.21 ± 0.09 | 1.39 ± 0.09 | 1.18 ± 0.08 |
| *Gammacoronavirus* | IBV (Kr/D49/20) | 0.89 ± 0.04 | 0.91 ± 0.01 | 1.03 ± 0.17 | 1.60 ± 0.30 | 1.27 ± 0.11 | 1.80 ± 0.11 |
| *Deltacoronavirus* | PDCoV (KNU1607) | 0.82 ± 0.06 | 0.96 ± 0.07 | 1.15 ± 0.07 | 1.42 ± 0.1 | 1.16 ± 0.21 | 1.51 ± 0.08 |

[1]PEDV, porcine epidemic diarrhea virus; SARS-CoV-2, severe acute respiratory syndrome coronavirus 2; BCoV, bovine coronavirus; IBV, infectious bronchitis virus; PDCoV, porcine deltacoronavirus.

**Supplementary Table S12. The selectivity index (SI) of Atglistatin and its derivatives for coronaviruses**

| Genus | Virus (strain)[1] | SI | | | | | |
|---|---|---|---|---|---|---|---|
| | | DABPU-DE | DABPU-EM | DABPU-DM (Atglistatin) | DABPU-Morph | DABPU-Pyrrol | DABPU-DPh |
| *Alphacoronavirus* | PEDV (QIAP1401) | 363.8 ± 14.94 | 279.8 ± 4.37 | 300.6 ± 23.77 | 148.4 ± 10.58 | 87.9 ± 5.51 | 106.5 ± 5.84 |
| *Betacoronavirus* | SARS-CoV-2 (KCDC03) | 848.8 ± 51.3 | 759.4 ± 65.68 | 629.1 ± 44.79 | 395.9 ± 40.20 | 197.2 ± 20.85 | 146.8 ± 12.28 |
| | SARS-CoV-2 (KDCA51463) | 757.0 ± 40.89 | 648.3 ± 48.02 | 588.0 ± 51.81 | 330.9 ± 28.50 | 189.9 ± 17.12 | 180.0 ± 15.75 |
| | SARS-CoV-2 (KDCA55905) | 875.3 ± 54.54 | 664.5 ± 50.43 | 530.4 ± 52.54 | 348.1 ± 25.71 | 307.6 ±14.63 | 206.1 ± 11.01 |
| | SARS-CoV-2 (Omicron, BA5) | 965.9 ± 66.34 | 565.5 ± 36.68 | 491.8 ± 27.72 | 272.8 ± 16.42 | 155.4 ± 16.20 | 168.2 ± 15.02 |
| *Betacoronavirus* | BCoV (KWD20) | 366.6 ± 16.41 | 349.0 ± 25.19 | 318.3 ± 12.94 | 149.8 ± 11.51 | 103.8 ± 7.16 | 92.1 ± 6.93 |
| *Gammacoronavirus* | IBV (Kr/D49/20) | 284.8 ± 15.12 | 273.6 ± 4.52 | 240.29 ± 39.10 | 103.7 ± 19.50 | 102.6 ± 8.68 | 61.6 ± 4.56 |
| *Deltacoronavirus* | PDCoV (KOR/KNU1607) | 322.0 ± 22.48 | 268.5 ± 19.83 | 219.9 ± 13.74 | 135.8 ± 9.67 | 107.9 ± 19.58 | 74.8 ± 4.57 |

[1]PEDV, porcine epidemic diarrhea virus; SARS-CoV-2, severe acute respiratory syndrome coronavirus 2; BCoV, bovine coronavirus 3; IBV, infectious bronchitis virus 4; PDCoV, porcine deltacoronavirus.

**Supplementary Table S13. The combination index (SI) and drug reduction index of DABPU-DE and remdesivir combination for SARS-CoV-2 in Vero E6 cells**

| DABPU-DE (µM) | Remdesivir (µM) | Fraction affected (Fa) | Combination index (CI) |
|---|---|---|---|
| 0.06 | 0.09 | 0.85 | 0.38±0.10 |
| 0.12 | 0.09 | 0.82 | 0.51±0.18 |
| 0.25 | 0.09 | 0.86 | 0.58±0.199 |
| 0.5 | 0.09 | 0.93 | 0.57±0.01 |
| 1.0 | 0.09 | 0.91 | 1.0±0.12 |
| 0.06 | 0.18 | 0.88 | 0.62±0.09 |
| 0.12 | 0.18 | 0.82 | 0.85±0.01 |
| 0.25 | 0.18 | 0.9 | 0.71±0.30 |
| 0.5 | 0.18 | 0.94 | 0.67±0.07 |
| 1.0 | 0.18 | 0.9 | 0.94±0.13 |
| 0.06 | 0.35 | 0.96 | 0.56±0.29 |
| 0.12 | 0.35 | 0.98 | 0.43±0.19 |
| 0.25 | 0.35 | 0.98 | 0.47±0.22 |
| 0.5 | 0.35 | 0.98 | 0.58±0.16 |
| 0.25 | 0.75 | 0.99 | 0.53±0.11 |
| 0.5 | 0.75 | 0.99 | 0.40±0.31 |

## References


1. **Amer, H.M., Almajhdi, F.N., 2011.** Development of a SYBR Green I based real-time RT-PCR assay for detection and quantification of bovine coronavirus. Mol. Cell. Probes 25, 101-107. https://doi.org/10.1016/j.mcp.2011.03.001.
2. **Baek Y.B., Kwon, HJ., Sharif, M., Lim, J., Less, I.C., Ryu, Y.B., et al., 2022.** Therapeutic strategy targeting host lipolysis limits infection by SARS-CoV2 and influenza A virus. Signal Thransduct. Target. Ther. 7,367. https://doi.org/10.1038/s41392-022-01223-4.
3. **Deng, X., Buckley, A.C., Pillatzki, A., Lager, K.M., Baker, S.C., Faaberg, K.S., 2021.** Development and utilization of an infectious clone for porcine deltacoronavirus strain USA/IL/2014/026. Virology 553, 35-45. https://doi.org/10.1016/j.virol.2020.11.002.
4. **Farooq, M., Ali, A., Hassan, M. S., Abdul-Careem, M. F., 2024.** Nucleotide and amino acid analyses of unique infectious bronchitis virus (IBV) variants from Canadian poultry flocks with drop in egg production. https://doi.org/10.3390/genes15111480.
5. **Kim, Y., Oh, C., Shivanna, V., Hesse, R.A., Chang, K.O., 2017.** Trypsin-independent porcine epidemic diarrhea virus US strain with altered virus entry mechanism. BMC Vet. Res. 13, 1-13. https://doi.org/10.1186/s12917-017-1283-1.
6. **Ogando, N.S., Dalebout, T.J., Zevenhoven-Dobbe, J.C., Limpens, R.W.A.L., van der Meer, Y., Caly, L., et al., 2020.** SARS-coronavirus-2 replication in Vero E6 cells: replication kinetics, rapid adaptation and cytopathology. J. Gen. Virol. 101, 925-940. https://doi.org/10.1099/jgv.0.001453.
7. **Truong, A.D., Park, B., Ban, J., Hong, Y.H., 2016.** The novel chicken interleukin 26 protein is overexpressed in T cells and induces proinflammatory cytokines. Vet. Res. 47, 65. https://doi.org/10.1186/s13567-016-0342-0.
8. **Wang, B., Liu, Y., Ji, C. M., Yang, Y. L., Liang, Q. Z., Zhao, P., ... & Huang, Y. W. (2018).** Porcine deltacoronavirus engages the transmissible gastroenteritis virus functional receptor porcine aminopeptidase N for infectious cellular entry. *Journal of virology*, *92*(12), 10-1128. https://doi.org/10.1128/jvi.00318-18.
